# *In vitro*, *in planta,* and comparative genomic analyses of *Pseudomonas syringae* pv. *syringae* strains of pepper (*Capsicum annuum* var. *annuum*)

**DOI:** 10.1128/spectrum.00064-24

**Published:** 2024-05-07

**Authors:** Sochina Ranjit, Loïc Deblais, Jelmer W. Poelstra, Menuka Bhandari, Francesca Rotondo, Joy Scaria, Sally A. Miller, Gireesh Rajashekara

**Affiliations:** 1 Department of Animal Sciences, The Ohio State University, Wooster, Ohio, USA; 2 Molecular and Cellular Imaging Center, Wooster, Ohio, USA; 3 Department of Plant Pathology, The Ohio State University, Wooster, Ohio, USA; 4 Department of Veterinary Pathobiology, Oklahoma State University, Stillwater, Oklahoma, USA; San Joaquin Valley Agricultural Sciences Center, Parlier, California, USA

**Keywords:** *Pseudomonas syringae *pv. *syringae*, phytobacteria, pepper plants, virulence genes, whole-genome sequence analysis, comparative genomic analysis

## Abstract

*Pseudomonas syringae* pv. *syringae* (*Pss*) is an emerging phytopathogen that causes *Pseudomonas* leaf spot (PLS) disease in pepper plants. *Pss* can cause serious economic damage to pepper production, yet very little is known about the virulence factors carried by *Pss* that cause disease in pepper seedlings. In this study, *Pss* strains isolated from pepper plants showing PLS symptoms in Ohio between 2013 and 2021 (*n* = 16) showed varying degrees of virulence (*Pss* populations and disease symptoms on leaves) on 6-week-old pepper seedlings. *In vitro* studies assessing growth in nutrient-limited conditions, biofilm production, and motility also showed varying degrees of virulence, but *in vitro* and *in planta* variation in virulence between *Pss* strains did not correlate. Comparative whole-genome sequencing studies identified notable virulence genes including 30 biofilm genes, 87 motility genes, and 106 secretion system genes. Additionally, a total of 27 antimicrobial resistance genes were found. A multivariate correlation analysis and Scoary analysis based on variation in gene content (*n* = 812 variable genes) and single nucleotide polymorphisms within virulence genes identified no significant correlations with disease severity, likely due to our limited sample size. In summary, our study explored the virulence and antimicrobial gene content of *Pss* in pepper seedlings as a first step toward understanding the virulence and pathogenicity of *Pss* in pepper seedlings. Further studies with additional pepper *Pss* strains will facilitate defining genes in *Pss* that correlate with its virulence in pepper seedlings, which can facilitate the development of effective measures to control *Pss* in pepper and other related *P. syringae* pathovars.

**IMPORTANCE:**

*Pseudomonas* leaf spot (PLS) caused by *Pseudomonas syringae* pv. *syringae* (*Pss*) causes significant losses to the pepper industry. Highly virulent *Pss* strains under optimal environmental conditions (cool–moderate temperatures, high moisture) can cause severe necrotic lesions on pepper leaves that consequently can decrease pepper yield if the disease persists. Hence, it is important to understand the virulence mechanisms of *Pss* to be able to effectively control PLS in peppers. In our study, *in vitro*, *in planta*, and whole-genome sequence analyses were conducted to better understand the virulence and pathogenicity characteristics of *Pss* strains in peppers. Our findings fill a knowledge gap regarding potential virulence and pathogenicity characteristics of *Pss* in peppers, including virulence and antimicrobial gene content. Our study helps pave a path to further identify the role of specific virulence genes in causing disease in peppers, which can have implications in developing strategies to effectively control PLS in peppers.

## INTRODUCTION

*Pseudomonas syringae* pv. *syringae* (*Pss*) is classified as a pathovar of the *P. syringae* complex, which is notorious for causing diseases in a wide and often overlapping range of host plants from Solanaceae and Leguminosae plants to citrus and stone fruit trees (1). *Pss* is an emerging pathogen that causes *Pseudomonas* leaf spot (PLS) disease in peppers (*Capsicum annuum* var. *annuum*). Peppers are an important annual crop used for fresh market consumption and processed products (2). In 2020, 4.7 million pounds of peppers were produced in the US, valued at $579 million (3). However, PLS can be a serious disease in pepper plants (4, 5), which can significantly reduce the yield and quality of crops due to the formation of severe dark necrotic lesions on pepper foliage. Due to a lack of knowledge on the virulence mechanisms and pathogenicity of *Pss* in pepper, *Pss* is difficult to control once it establishes in the field.

Studies have shown that *Pss,* in other plant hosts such as cherry trees, mango trees, and bean plants, has a repertoire of virulence mechanisms that allow it to persist on seeds over winter, survive harsh abiotic stressors, and colonize the apoplast to cause disease in plants (6–8). First, biofilm formation via alginate biosynthesis gene cluster aids *Pss* in surviving abiotic stressors (9). Second, flagellar and twitching motility genes help *Pss* move from the outside to the inside of the leaf (10). Finally, secretion system gene clusters (types I–VI), which encode proteins that weaken and invade the host immune defenses, help *Pss* establish as a pathogen in the host plant (11). *Pss* uses host defense and stress response gene products to evade, survive, and cause disease in host cells. Currently, copper-based (copper sulfate or copper hydroxide) or streptomycin-based antimicrobials are used as chemical control methods for *Pss* in peppers (12, 13). However, in recent years, the intensive application of copper-based antimicrobials to reduce PLS during the growing season has resulted in the emergence of copper-resistant strains of *Pss,* making copper-based antimicrobial agents less effective (8, 14).

Understanding the pathogenicity, virulence mechanisms, and behavior of *Pss* in peppers is needed for the prudent use of antimicrobials to mitigate PLS in peppers. Not only has the application of antimicrobials been an insufficient control method, but it has also given rise to antimicrobial resistance genes in plant agriculture and created resistant phytopathogens. This serves as a precautionary tale to understand the pathogen at hand before exploiting intensive control methods. Given the lack of effective management of PLS and the knowledge gap about virulence factors of *Pss* expressed in pepper seedlings, there is a need to explore the gene content of *Pss* in peppers so that more effective and sustainable management techniques can be developed. *Pss* causing PLS in peppers was reported as early as 1962 in the US (15), yet there is no published research on the virulence genes present in *Pss* specifically affecting pepper plants.

The purpose of this study was to investigate differences in virulence of *Pss* strains *in vitro* (growth rate, motility, and biofilm production), in pepper seedlings (*Pss* population and disease symptoms on leaves), and through whole-genome sequence analysis (functional genomics and non-synonymous single nucleotide variations). The genomic study gave insight into genomic features as well as the virulence and antimicrobial gene profile of the *Pss* strains that cause disease in pepper seedings. Overall, this study, for the first time, provides insights into *in vitro* and *in planta* characteristics of *Pss* in pepper as well as the virulence and antimicrobial resistance gene content of *Pss* that causes disease in pepper.

## RESULTS

### Characterization of *Pss* strains

A total of 16 *Pss* strains were isolated from pepper plants harboring characteristic PLS symptoms. These samples came from three different counties in northern Ohio between 2013 and 2021 (Table S1). The 16 strains were fluorescent on *Pseudomonas* F agar, oxidase-negative, arginine dihydrolase-negative, and levan-positive on NSA medium, induced a hypersensitive response (HR) 24 h post-infection on tobacco (*Nicotiana tabacum*) leaves cv. “Samsun,” and did not cause soft rot on potato slices (Table S1). These features indicate that the strains belong to the LOPAT group Ia, which is characteristic of *P. syringae* pathovars (16). Further, PCR assays using two primers (*hrpZ* and *syrB* genes) identified the 16 isolates as being *Pss* strains (Table S1). The *hrpZ* (encoding for a T3SS hypersensitive response and pathogenicity protein) primer is commonly used as a species-specific primer for *Pseudomonas syringae* (17), and the *syrB* (encoding for a protein involved in the biosynthesis of syringomycin toxin) primer has recently been shown to be a pathovar-specific primer for *Pss* (18).

### PLS symptoms on pepper seedlings and *Pss* bacterial populations varied by *Pss* strain

**Fig 1:**
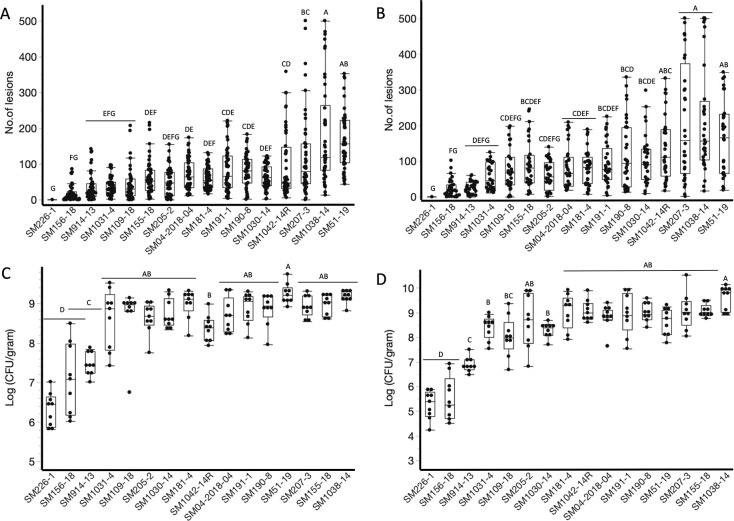
Disease severity and bacterial populations on “California Wonder” pepper seedlings 3 and 7 dpi with 16 *Pss* strains. Disease severity is represented by the number of lesions on two lower leaves of infected seedlings at 3 dpi (**A**) and 7 dpi (**B**) and populations of *Pss* strains in terms of log CFU/g at 3 dpi (**C**) and 7 dpi (**D**). The experiment was conducted twice with a minimum of four replicates for each strain at different time points, and data from the two experiments were combined. The black bars represent the median number of lesions or log (CFU/g). Different letters represent strains that are significantly different (*P* < 0.05), and the error bars represent the standard deviation.

The disease severity (number of lesions on the surface of two lower leaves for each seedling) was recorded at 3, 7, and 14 days post-infection (dpi). The disease incidence (number of seedlings showing PLS symptoms) was recorded at 3, 7, and 14 dpi. The bacterial load in inoculated seedlings was determined at 0, 3, 7, and 14 dpi using the direct dilution plating method. All *Pss* strains but SM226-1 caused characteristic PLS symptoms on inoculated pepper leaves 3, 7, and 14 dpi. A gradual increase in disease severity was observed over time (average number of lesions of 73.2 ± 54.3 at 3 dpi, 96.1 ± 68.0 at 7 dpi, and 245.5 ± 122.8 at 14 dpi). At 3 dpi, strains SM51-19 and SM1038-14 caused the highest disease severity (*P* < 0.05) with mean lesion counts of 169.9 ± 85.2 and 186.0 ± 147.9, respectively. Most strains (*n* = 9/16) caused moderate disease severity, with the number of lesions ranging from 49.8 ± 39.7 to 94.1 ± 90.1 (*P* < 0.05). Four strains caused low disease severity on pepper seedlings with the number of lesions ranging from 16.1 ± 12.0 to 41.3 ± 34.7 (Fig. 1A, *P* < 0.05). Only one *Pss* strain (SM226-1) did not cause disease symptoms on pepper foliage (*P* < 0.05).

Disease severity increased overall from 3–7 dpi. At 7 dpi, disease severity was highest for plants inoculated with strains SM1042-14R, SM51-19, SM207-3, and SM1038-14 with lesion counts of 136.2 ± 90.2, 168.0 ± 103.85, 213.1 ± 166.7, and 213.8 ± 151.3, respectively (Fig. 1B). Six strains caused moderate disease severity, with the mean number of lesions ranging from 81.0 ± 49.0 to 117 ± 96.4. Strains SM226-1, SM156-18, SM1031-4, SM1031-4, SM205-2, and SM109-18 caused the lowest disease severity with lesion counts of 0, 22.6 ± 17.5, 26.2 ± 16.8, 56.1 ± 36.4, 60.0 ± 4, and 75.3 ± 56.5, respectively. Three *Pss* strains (SM207-3, SM51-19, and SM914-13) showed marginally lower disease severity at 7 dpi compared to 3 dpi; this decrease in disease severity might be due to differences in the genomic makeup of these strains, where some strains appear to have better adapted to pepper plants in surviving and causing disease, while other strains initially cause disease but later decrease in population count, resulting in a lower number of lesions at later time points (Fig. 1). By 14 dpi, most strains (*n* = 12/16) caused high disease severity with lesion counts ranging from 109.6 ± 43.58 to 409.6 ± 112.74 (Fig. S1, *P* < 0.05). Strains SM190-8, SM914-13, SM156-18, and SM226-1 caused no or low disease severity at 14 dpi with lesion counts ranging from 0 to 17.4 ± 8.64 (Fig. S1, *P* < 0.05).

Similar trends were observed for bacterial populations on pepper seedlings inoculated with *Pss* strains at 3 and 7 dpi (Fig. 1C and D). However, drastic reductions in bacterial loads were observed at 14 dpi due to high disease progression causing leaf senescence (data not shown). At 3 dpi, with the exception of SM226-1, SM156-18, and SM914-13, population counts from inoculated pepper seedlings did not differ significantly among *Pss* strains, with average bacterial loads in the range of 8.4 ± 0.3 log CFU/g to 9.2 ± 0.1 log CFU/g (Fig. 1C; *P* < 0.05). Pepper seedlings inoculated with strains SM156-18 and SM914-13 harbored significantly lower bacterial populations than those inoculated with any of the 13 more virulent strains, with an average bacterial load of 7.1 ± 0.3–7.5 ± 0.9 log CFU/g. Although seedlings inoculated with SM226-1 were asymptomatic, they yielded an average bacterial population of 6.3 ± 0.4 log CFU/g (*P* < 0.05). At 7 dpi (Fig. 1D), average bacterial loads in pepper seedlings for the 16 *Pss* strains ranged from 5.2 ± 0.6 to 9.6 ± 0.5 log CFU/g. *Pss* populations by strain ranked in similar order at 3 and 7 dpi.

In addition, significant differences in disease incidence (number of seedlings showing PLS symptoms) were observed between the strains at 3, 7, and 14 dpi (Fig. S2). SM226-1 was the only *Pss* strain that did not cause disease at any time point (Fig. S2). All 16 *Pss* strains showed increased disease incidence over time from 3 to 14 dpi. At 3 dpi, disease incidence was high in most strains (*n* = 15/16, average disease incidence of 98.6% ± 2.7%; *P* < 0.05). At 7 dpi, the disease incidence increased with most strains (*n* = 15/16, average disease incidence of 99.0% ± 1.3%; *P* < 0.05). At 14 dpi, incidence further increased in most strains (*n* = 15/16, average disease incidence of 99.8% ± 0.8%; *P* < 0.05) (Fig. S2).

### Growth, motility, and biofilm formation of the *Pss* strains

**Fig 2:**
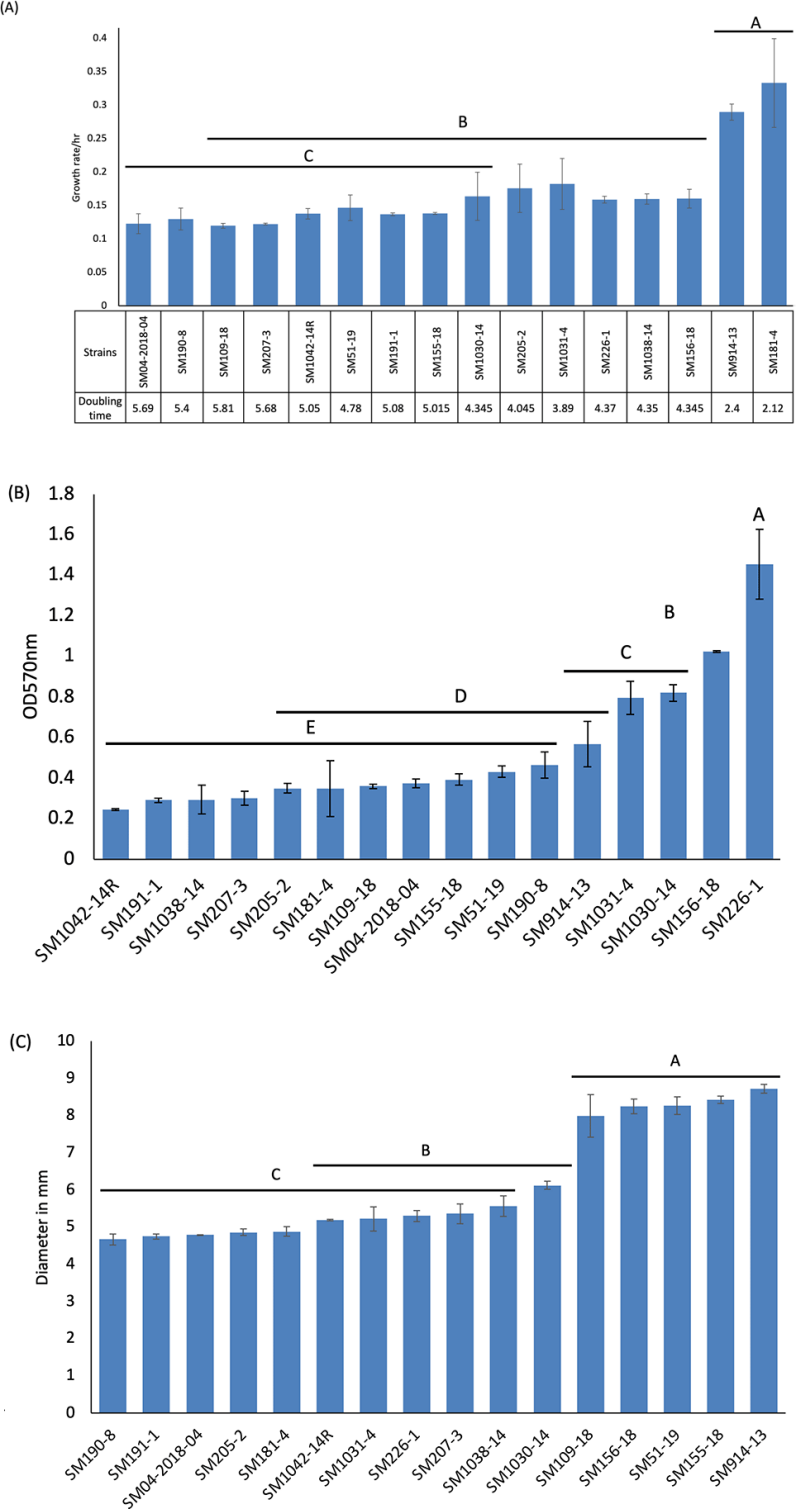
*In vitro* (growth, biofilm, and motility) characteristics of 16 *Pss* strains. (**A**) The growth rate and doubling time of *Pss* strains, calculated using the growthcurver 0.3.1 package in R 4.3.1. The OD_600_ measurement of *Pss* strains taken over 24 h in M9 media was used in growthcurver 0.3.1 in R to calculate the growth rate and doubling time. (**B**) Quantity of biofilm produced by *Pss* strains after 72 h of incubation at 28°C in M9 minimal broth. OD_570_ was measured to quantify biofilm production. (**C**) Motility of *Pss* strains was measured after 24 h incubation on Luria–Bertani (LB) semi-solid agar medium (0.3%) at 28°C. Motility was determined by measuring the diameter (in millimeter) of the halo. Each of the *in vitro* experiments was conducted twice with three replicates each. The bars represent standard error, and the different letters represent strains that are significantly different (*P* < 0.05).

Growth, biofilm formation, and motility in nutrient-limited conditions were determined (Fig. 2) to understand how these phenotypes correlate with the observed variation in *Pss* population and disease severity in pepper seedlings. Different growth patterns were observed between the *Pss* strains in M9 minimal broth over 48 h at 28°C (Fig. 2A; *P* < 0.05). SM181-4 and SM914-13 had the significantly highest growth rate whereas SM190-8 and SM04-2028-04 had the lowest growth rates (Fig. 2A, *P* < 0.05). The strains with the highest growth rates *in vitro* did not have the highest disease severity *in planta* (Fig. 1).

All *Pss* strains produced biofilm that was measured after 72 h at 28°C in nutrient-limiting M9 minimal broth. However, the strains varied in the quantity of biofilm produced (Fig. 2B). Strain SM226-1 produced significantly (*P* < 0.05) more biofilm [average optical density (OD)_]570_ 1.4 ± 0.2] than all other strains. Strains SM1031-4, SM1030-14, and SM156-18 produced similar amounts of biofilm (average OD_570_ 1.0 ± 0, 0.8 ± 0.03, and 0.8 ± 0.06, *P* < 0.05). The remaining strains produced relatively low quantities of biofilm. Similar to the growth rate trends, the strains that produced the highest quantities of biofilm *in vitro* did not have the highest disease severity *in planta*. In fact, the highest biofilm-producing strain (SM226-1) caused no disease symptoms *in planta* at 3 dpi although an average of 6.3 ± 0.4 log CFU/g of the bacterial population was detected (Fig. 1).

Motility (swarming) of the *Pss* strains was assessed in a semi-solid LB agar (0.3%) medium for 24 h at 28°C. All *Pss* strains were motile (Fig. 2C); however, the motility of *Pss* strains did not follow the patterns observed during the growth and biofilm assays. Strains SM109-18, SM156-18, SM51-19, SM155-18, and SM914-13 were significantly more motile [average diameter of the halo (mm) of 7.9 ± 0.02, 8.2 ± 0.01, 8.3 ± 0.06, 8.4 ± 0.04, and 8.7 ± 0.2; *P* < 0.05] compared to the other remaining 11 *Pss* strains, which had relatively low motility [average diameter of halo (mm) between 4.7 ± 0.01 and 6.1 ± 0.01; *P* < 0.05]. Except for SM51-19, the strains that were highly motile did not induce the most severe disease *in planta*. In fact, the moderately motile strains SM226-1 and SM207-3 caused, respectively, no symptoms and high disease severity, indicating that motility measured *in vitro* could not explain the variation in pathogenicity of *Pss* strains seen *in planta*.

Additionally, a linear regression analysis between *in vitro* data (growth, biofilm, and motility) and *in planta* data (disease severity) only showed a significant correlation between biofilm and disease severity at 3 dpi (*r*^2^ = 0.37, *P* < 0.05) and 7 dpi (*r*^2^ = 0.38, *P* < 0.05). The results of the regression analysis indicated that there was no significant relationship (*P* > 0.05) between the growth or motility of *Pss* strains and disease severity on 3 and 7 dpi. The multivariate correlation analysis conducted with a Benjamini–Hochberg (BH) correction showed that none of the genes were significantly correlated. Scoary detected a total of 31 genes (*n* = 1, 3, and 28 for growth, biofilm, and motility, respectively) that were associated with *in vitro* characteristics. However, after the Benjamini–Hochberg correction, none of the genes were significantly associated (Table S2).

### Genome characteristics and phylogenetic relationships of the 16 *Pss* strains

**Fig 3:**
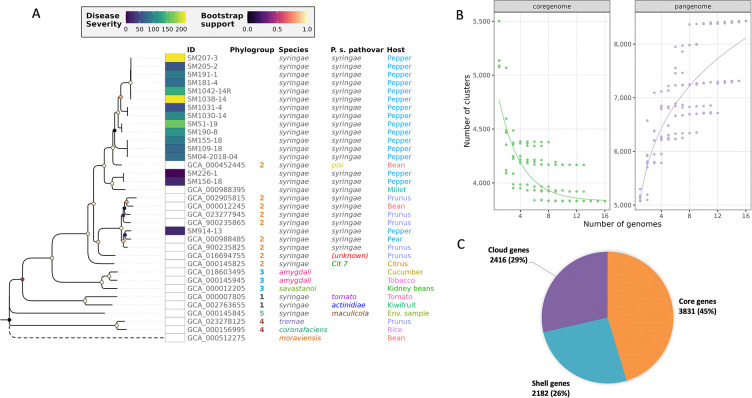
Phylogenetic, core, and pangenome analysis of 16 *Pss* strains. (**A**) Whole-genome core SNP-based phylogeny of phytopathogenic *Pseudomonas* strains (*n* = 34). K-chooser was used to find the optimal k-mer (19-mer), and the core SNPs were calculated by KSNP3. The maximum likelihood phylogeny tree was plotted in R. The branch length of the tree correlates with the SNP distance. Each pathovar as well as host/source of isolation is indicated by a different color. Pepper pathovars colored in blue are strains collected and used in this study. The branch for *Pseudomonas moraviensis* used as the outgroup species is truncated as indicated by the dashed line. The disease severity of the 16 *Pss* strains is indicated with a color spectrum, where dark blue indicates low disease severity and yellow indicates high disease severity. The bootstrap support for the branches is indicated with a color spectrum, where dark purple indicates low bootstrap support and light yellow indicates high bootstrap support. (**B**) Pie chart showing the pangenome composition of the 16 *Pss* strains. Core genes are present in 100% of the strains, shell genes in 15%–99%, and cloud genes in <15% of strains. (**C**) Rarefaction curves for core and pangenome of the 16 *Pss* strains. The lefthand plot shows how the size of the core genome (*y*-axis) decreases with the addition of more genome assemblies (*x*-axis), with individual points showing the core genome size for each possible combination of genomes for each value of *x*, the number of included genomes, and the line showing the fitted line following an exponential decay function. The righthand plot is similar but shows the increase of the pangenome with an increasing number of genomes, and the fitted line follows a power-law distribution.

The average genome assembly size of the 16 *Pss* genomes was 6.04 ± 0.10 Mbp with an average GC content of 59.12% ± 0.27%. The average N50 contig length was 229.6 ± 104.9 kbp, and the average number of contigs was 147 ± 72 (Table 1). Human contaminant contigs were identified by running Kraken2 v.2.1.2 (19) with the Standard Plus Fungi database downloaded from https://benlangmead.github.io/aws-indexes/ on 8 April 2022 and option “—confidence 0.5,” and then, the contaminant contigs were removed from our assemblies using the extract_kraken_reads.py script from KrakenTools v.1.2 (20). The average depth of coverage was 57% ± 8.26%. A phylogenetic analysis using kSNP3 was conducted to determine the relatedness of the 16 *Pss* strains to other *P. syringae* strains (*n* = 18) downloaded from NCBI both within and outside the *P. syringae* pv. *syringae* phylogenetic group (Fig. 3A; Tables S1 and S3). A total of 936,714 single nucleotide polymorphisms (SNPs) were identified across the 34 genomes, 1,845 of which were core (shared) SNPs. Based on the core SNPs, a phylogenetic tree with all 34 genomes was generated (Fig. 3A). *Pss* strains (*n* = 15/16) sequenced in this study formed a clade including a *Pseudomonas syringae* pv. *pisi* strain isolated from beans and a *Pseudomonas syringae* pv. *syringae* strain from millet with a bootstrap value of 0.927. The SM914-13 strain was the only strain outside of this clade grouped with the other *Pss* and non-*Pss* strains from NCBI.

**TABLE 1:** Genome assembly statistics of *Pss*

Sample number	Genome size	Number of contigs	GC (%)	Bacterial BUSCO	N50	Completeness^*a*^	Contamination	Number of predicted genes
SM04-2018-04	5,960,405	79	59.17	100	534,491	100	0.11	5,108
SM1030-14	6,135,699	152	58.98	99.32	193,480	99.68	0.43	5,300
SM1031-4	6,159,242	293	58.98	99.32	145,934	99.68	0.43	5,370
SM1038-14	6,045,224	132	58.97	99.32	205,021	100	0.11	5,166
SM1042-14R	6,057,882	235	59.01	100	129,352	100	0.47	5,272
SM109-18	5,964,538	95	59.17	100	350,263	100	0.6	5,117
SM155-18	5,954,342	61	59.17	100	339,735	100	0.14	5,103
SM156-18	5,982,996	307	59.08	99.32	112,094	99.68	0.47	5,303
SM181-4	6,026,070	110	59	100	193,779	100	0.43	5,165
SM190-8	5,951,629	95	59.11	100	275,109	100	0.43	5,104
SM191-1	6,034,096	124	58.99	99.32	205,037	100	0.43	5,171
SM205-2	6,026,772	112	59.01	99.32	164,342	100	0.43	5,166
SM207-3	6,026,178	100	59.01	100	205,037	100	0.43	5,155
SM226-1	5,956,914	185	59.07	99.32	146,860	99.68	0.11	5,216
SM51-19	5,953,602	92	59.1	100	305,681	100	0.6	5,101
SM914-13	6,326,320	185	59.14	98.65	208,630	100	0.43	5,585

^*a*^ The mean completeness based on the presence of universal genes that are present in the sequenced genomes that are expected to be in the genomes was 99.90% ± 0.152%.

### Gene content and pangenome analysis

An analysis comparing the gene content among the 16 *Pss* strains using Roary v.3.11.2 (21) following annotation with Prokka v.1.14.6 (22) identified a total of 8,429 genes, of which 3,831 (45.5%) were core genes (defined as genes present in at least 99% of genomes; in our case, all genomes), 0 were soft core genes (present in 95%–99% of genomes), 2,182 (25.9%) were shell genes (present in 15%–95% of genomes), and 2,416 (28.7%) were cloud genes (present in <15% of genomes) (Fig. 3B). The rarefaction curves for the pangenome showed that the plot came close to plateauing (Fig. 3C). Heaps’ law used to determine the openness of the pangenome showed that pangenome was closed with a marginal alpha value of 1.000004. Automatic genome annotation using the Rapid Annotations using Subsystems Technology toolkit (RAST) and SEED (23) predicted an average of 5,223 ± 132 coding sequences among the 16 *Pss* strains. Within these coding sequences, there was an average of 361.3 ± 4.2 subsystems (subsystems in RAST are used to organize related functional roles of genes into categories that have specific biological functions) (23) among the 16 *Pss* strains containing essential bacterial genes and genes associated with virulence. The virulence-associated genes were related to motility and chemotaxis, secretion systems, invasion and intracellular resistance, iron acquisition, biofilm, stress response, detoxification, bacteriocins, and antibiotic resistance.

### Multidrug resistance genes including copper resistance genes

After the annotation of antibiotic resistance genes using the *Pseudomonas* genome database, ResFinder, CARD, NCBI, and ARG-ANNOT, a total of 27 antibiotic resistance genes were identified within the core and variable genes (Fig. S3). Most of these (*n* = 12/27) were associated with copper resistance, including genes associated with copper resistance proteins (*copBCD* and *copG*) and copper homeostasis and regulator proteins (*cutE* and *cusSR*). Other genes associated with copper resistance included copper ATPase, copper chaperone, multicopper oxidase, and cytochrome c heme (*ccmFH*) genes. There were several other heavy metal resistance proteins including the magnesium and cobalt resistance gene (*corC*), mercury resistance gene (*merR*), two genes associated with cadmium resistance [D-cysteine desulfydrase and Cd (II)/Pb (II) regulator], and a heavy metal resistance gene (*hmrR*). Several other antibiotic resistance genes (*n* = 7/27) were not associated with heavy metals, including streptothricin acetyltransferase (SAT) for streptothricin resistance (closely related to streptomycin), beta-lactamase C and metal-dependent hydrolase of beta-lactamase genes associated with beta-lactam resistance, penicillin-binding protein for resistance to penicillin, DNA gyrase AB associated with fluoroquinolone resistance, and *nfxB* gene associated with quinolone resistance. A multidrug resistance efflux gene (*cflA*) was also identified, which has been associated with resistance to various drugs like chloramphenicol.

### Plasmids and prophages

Two types of plasmids (KY362372 and CP006257) containing a total of nine genes were found among nine of the *Pss* strains (Table 2). These genes included two T4SS genes (*virB3* and *virD4*) located in the KY362372 plasmid. The plasmid KY362372 is one of the eight native *Pseudomonas syringae* plasmids that are part of the pPT23A family. KY362372 is known to carry genes important for virulence and epiphytic colonization of plant leaf surfaces (24). Both KY362372 and CP006257 plasmids have been identified in other *Pss* strains isolated from mangoes, pears, and millet (24–26). No prophages were identified in the *Pss* genomes by PHAge Search Tool Enhanced Release (PHASTER).

**TABLE 2:** Plasmids of *Pss* strains and the genes located in the plasmids^*a*^

Strain	Plasmid accession no.	Gene name	Virulence-associated
SM181-4	KY362372	Inner membrane protein forms channel for type IV secretion of T-DNA complex, VirB3	T4SS
SM109-18	CP006257	Hypothetical protein	NA
SM1038-14	KY362372	Hypothetical protein	NA
SM205-2	KY362372	Coupling protein VirD4, ATPase required for T-DNA transfer	T4SS
SM1042-14R	KY362372	Hypothetical protein	NA
SM914-13	KY362372	Error-prone, lesion bypass DNA polymerase V (UmuC)	NA
SM04-2018-04	CP006257	ISPpu14, transposase Orf2	NA
SM191-1	KY362372	Methyl-accepting chemotaxis protein I (serine chemoreceptor protein)	NA
SM207-3	KY362372	Hypothetical protein	NA

^*a*^ Two types of plasmids were found between nine *Pss* strains. Only two genes found in plasmid KY362372 were associated with the T4SS, which is a known virulence factor.

### Variable genome of *Pss* strains

**Fig 4:**
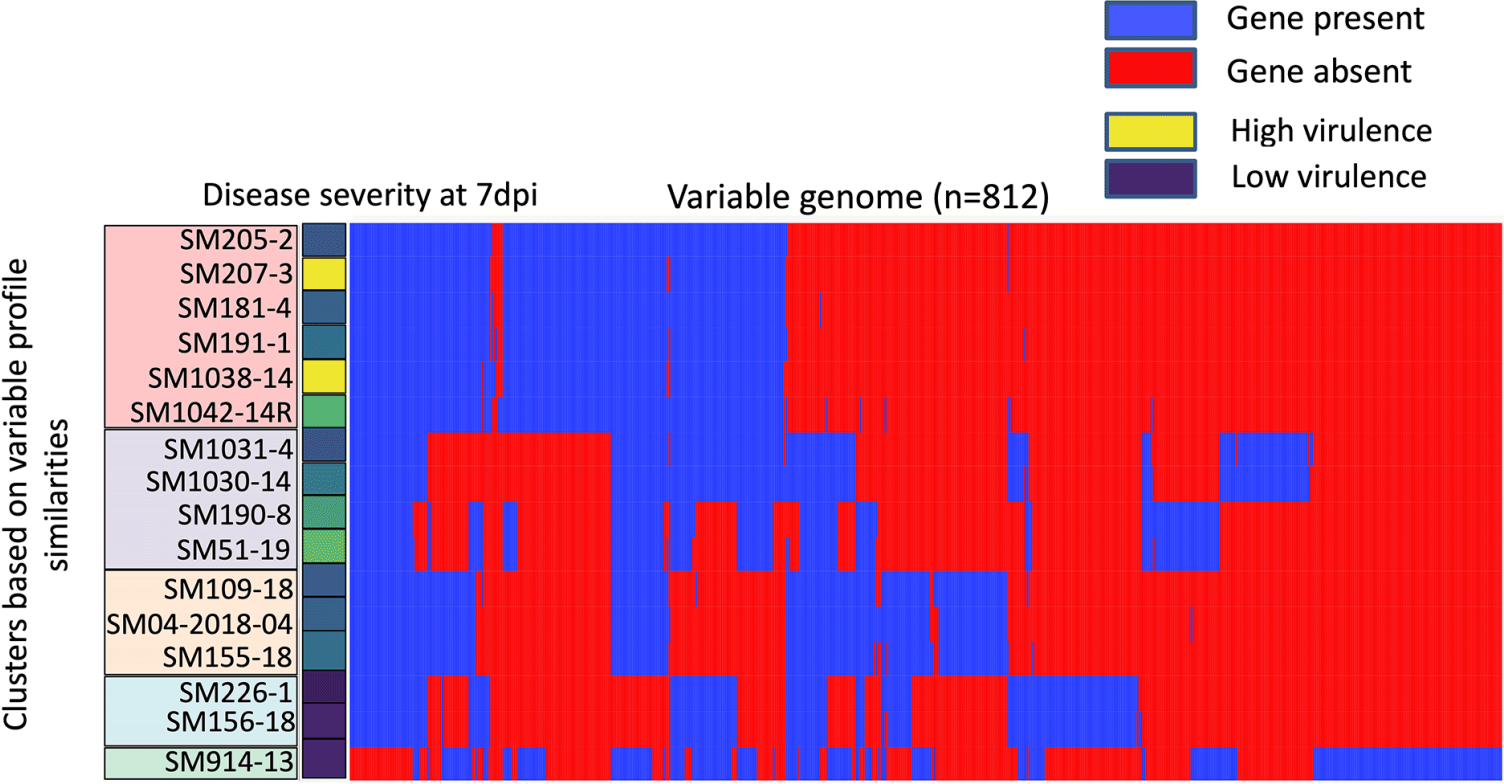
Hierarchical clustering based on presence/absence of variable genes (n=812) of *Pseudomonas syringae* pv*. syringae* (*Pss*) strains (n=16). On the left-hand side, colored boxes around the strain names indicate the cluster for each strain, and the disease severity score at 7 dpi is shown for each strain. On the right-hand side, each column represents a single gene with blue indicating presence of a gene, and red indicating absence of a gene.

**Fig 5:**
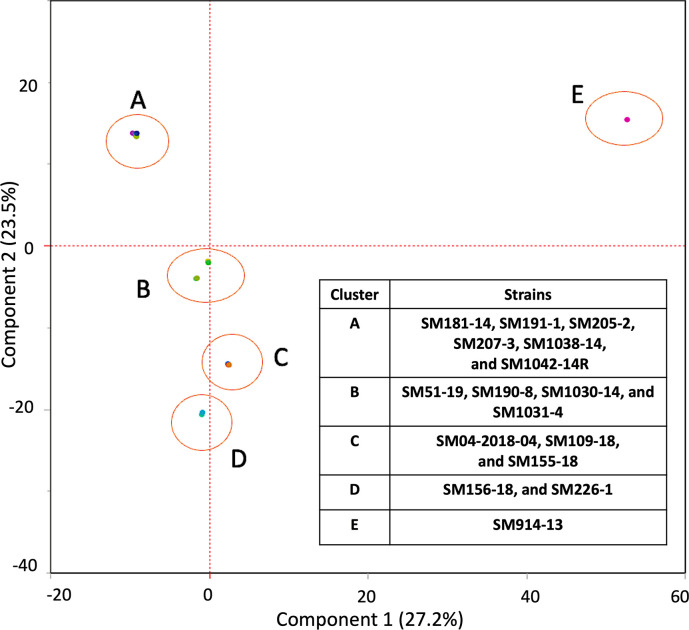
Principal component analysis (PCA) of variable genes (n=812) between *Pseudomonas syringae* pv*. syringae* (*Pss*) strains (n=16). Each dot represents the different *Pss* strains. PCA grouped the *Pss* strains into 5 clusters (A to E).

A total of 812 genes were identified in the variable genome among the 16 *Pss* strains (Fig. 4; Table S4). The variable gene profile of *Pss* strains causing high to moderate disease severity is distinct from the three strains (SM226-1, SM156-18, and SM914-13) causing low disease severity in pepper seedlings. Some of the variable genes present in *Pss* strains that cause high to moderate disease severity (lesion counts between 213.8 ± 151.3 and 56.1 ± 36.4) appear to be missing from the three strains (SM226-1, SM156-18, and SM914-13) causing low disease severity (lesion counts of 0, 22.6 ± 17.5, and 26.2 ± 16.8), indicating that variation in the gene content might explain the variation in virulence seen between the strains *in planta*. A principal component analysis (PCA) based on the variable genomes grouped the *Pss* strains into five clusters (*n* = 6, 4, 3, 2, and 1 strain in each cluster; Fig. 5). The four strains with high disease severity SM1042-14R, SM51-19, SM207-3, and SM1038-14 with lesion counts of 136.2 ± 90.2, 168.0 ± 103.85, 213.1 ± 166.7, and 213.8 ± 151.3, respectively, in pepper seedlings were grouped in Clusters A and B. Three strains with moderate disease severity (lesion counts between 75.3 ± 56.5 and 94.1 ± 90.1) were grouped in Cluster C. Two strains (SM226-1 and SM156-18) that had low disease (lesion counts of 0 and 22.6 ± 17.5) in pepper seedlings were grouped in Cluster D. SM914-13 (the sole member of Cluster E) had low disease severity in pepper seedlings (lesion counts of 26.2 ± 16.8).

### Motility, biofilm, secretion system, and antimicrobial resistance genes were identified in the pangenome and variable genome

**Fig 6:**
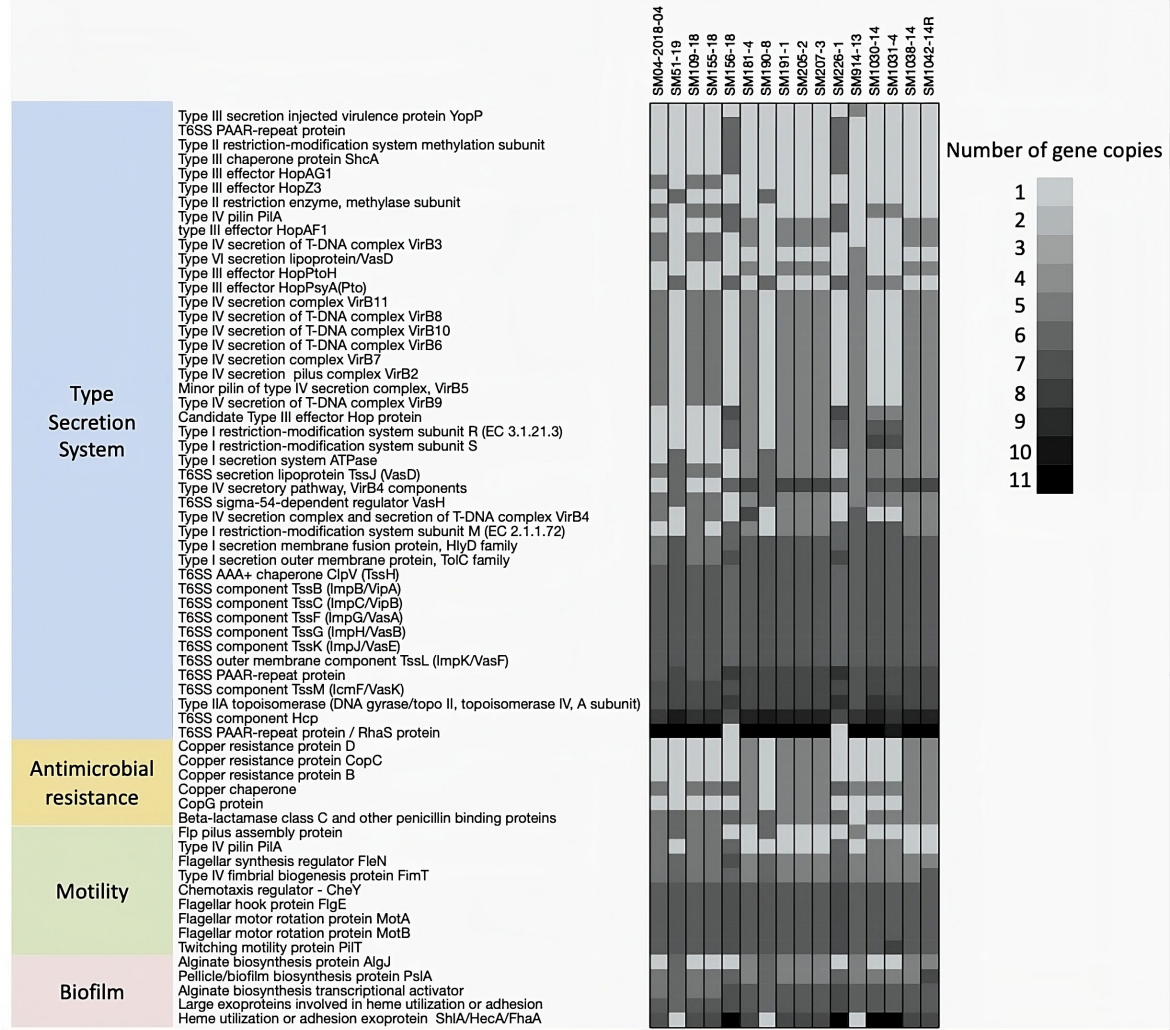
Virulence (secretion system, motility, and biofilm) and antimicrobial resistance genes of *Pseudomonas syringae* pv*. syringae* (*Pss*) strains (n=16) in the variable genome. A total of 29 secretion system genes, 2 motility genes, 2 biofilm genes and 6 antimicrobial resistance genes were found to be part of the variable genome. The darker grey boxes indicate more than one copy of the gene.

Virulence genes associated with motility, biofilm, and secretion system (*n* = 211; Fig. 6) were identified using ABRicate [against the Virulence Factor Database (VFDB)] and by comparing the functional annotations from RAST against the *Pseudomonas* genome database, Hop database, BastionHub database, and T3SEdb. The *Pss* strains contained a total of 87 motility genes that contribute to both flagellar and twitching motility (Fig. S4). Majority of the motility genes were associated with flagellar motility (*n* = 40/87). The flagellar motility genes corresponded to flagellar structure proteins, biosynthesis proteins, cap proteins, flagellar sensory and regularity proteins, and motor rotation proteins among other proteins. However, only two motility genes (*pilA* and *flp*) were part of the variable genome among the 16 *Pss* strains. These two genes were part of the twitching motility type IV pili assembly, and nearly half of the *Pss* strains (*n* = 7/16) carried these two genes (Fig. 6). The *Pss* strains contained a total of 30 biofilm genes that contribute to alginate and Psl polysaccharide biosynthesis and regulatory functions (Fig. S5). Majority of the biofilm genes were associated with alginate biosynthesis (*n* = 17/30), which included genes encoding for alginate biosynthesis two-component proteins, inner and outer membrane adhesion proteins, as well as transcriptional regulatory proteins. However, only two biofilm genes were part of the variable genome: the alginate biosynthesis gene *algJ* and adhesion-associated exoproteins (*shlA/hecA/fhaA*) genes (Fig. 6). Most strains carried the *algJ* (*n* = 9/16) and the *shlA/hecA/fhaA* (*n* = 13/16) genes. Interestingly, strains SM156-18, SM226-1, SM1030-14, and SM1031-4, carrying between five and nine copies of the *shlA/hecA/fhaA* genes, were also the highest biofilm producers (Fig. 6). In comparison, the other strains carried between 0 and 3 copies of the *shlA/hecA/fhaA* genes (Fig. 6).

A total of 106 secretion system genes were identified in the pangenome, which included 5 type I secretion system (T1SS), 3 type II secretion system (T2SS), 43 type III secretion system (T3SS), 34 type IV secretion system (T4SS), 1 type V secretion system (T5SS), and 18 type VI secretion system (T6SS) genes (Fig. S6). A total of 37 out of 106 genes associated with secretion systems (*n* = 2/5 genes for T1SS, *n* = 0/3 genes for T2SS, *n* = 17/43 T3SS, *n* = 13/34 for T4SS, *n* = 1/1 for T5SS, and *n* = 4/18 for T6SS) were identified as part of the variable genome (Fig. 6; Fig. S6). Strains SM156-18 and SM226-1 that caused low disease severity or no symptoms contained the lowest number of variable secretion system genes (*n* = 19/37) (Fig. 6). Most strains (*n* = 9/16) were missing some of the T3SS genes and carried the T4SS (*n* = 10/16) and T6SS (*n* = 14/16) genes. Eight genes were associated with T3SS including five T3SS effectors (*hopAG1, hopAF1, hopPtoH, hopPsyA*, and *hopZ3*), two T3SS virulence protein genes (*yopJ* and *yopP*), and a T3SS chaperone protein gene (*shcA*). Thirteen genes were associated with the T4SS including four inner membrane protein genes (*virB3, virB6, virB8*, and *virB10*), two ATPase (*virb4* and *virB11*), one outer membrane protein gene (*virB9*), two lipoprotein genes (*virB7* and *vasD*), two pilus genes (*virB2* and *virB5*), and one T4SS pilin gene *pilA*. For the T6SS, four genes were identified as variable among the 16 *Pss* strains. These genes were associated with the T6SS lipoprotein gene (*tssJ*), two PAAR repeat protein genes (*rhaS*), and a sigma-dependent regulator gene (*vasH*). Most *Pss* strains (*n* = 14/16) carried the *tssJ, rhaS,* and *vasH* genes. There was only one gene in the T1SS (type I secretion system ATPase) that was variable between the *Pss* strains. Most strains carried the variable T1SS gene (*n* = 11/16). The three identified T2SS genes (*pulE/tfp* pilus assembly pathway, ATPase *pilB*, and T2SS protein) were present among all *Pss* strains (*n* = 16/16). The only identified T5SS gene (channel forming *tpsB* family gene) was variable among the *Pss* strains. Most strains carried the variable T5SS gene (*n* = 13/16).

Only six out of 27 antimicrobial resistance genes were part of the variable genome. Most of the variability was found with the copper resistance genes (*n* = 5/6) including copper resistance proteins (*copBCD*, *copG*), copper tolerance, and copper chaperone genes (Fig. 6; Fig. S3). Fewer than half of the *Pss* strains (*n* = 6/16) carried the variable copper resistance genes. The only other variable gene was the beta-lactamase C and penicillin-binding protein, which was present in all but one *Pss* strain; SM914-13 was missing the resistance gene to beta-lactamase and penicillin.

### Gene and SNP association analyses did not produce significantly correlated genes

A multivariate correlation analysis and an association analysis with Scoary were performed to associate the presence of variable genes (*in silico*), non-synonymous SNPs within the variable genes, and pseudogenes (focusing on functional gene copies) with *in planta* phenotypes for all 16 *Pss* strains. The multiple correlation analysis and the Scoary run were conducted with Benjamini–Hochberg multiple-testing correction, which resulted in a lack of significant adjusted *P*-values (*P* > 0.05) for any genes obtained through the multivariate correlation analysis or Scoary (Tables S4 and S5; Fig. S7). A total of 142 variable genes had correlations with disease severity with uncorrected *P*-values below 0.05, many of which were positively correlated with secretion systems and biofilm genes (Table S4; Fig. S7). A total of 2,549 SNPs were identified in the virulence genes (motility, biofilm, and secretion system) of the 16 *Pss* genome assemblies using SM914-13 as the reference genome. After the multivariable correlation analysis, 30 SNPs primarily in the secretion system genes were correlated with disease severity (Table S6; Fig. S8). However, due to the small number of genomes analyzed (*n* = 16), significant correlations could not be detected in this study.

## DISCUSSION

*Pss* is known to infect numerous plant hosts (from stone fruit trees and legumes to Solanaceae crops) and causes economically important diseases in these crops (1). Peppers are one of the crops that can be infected by *Pss* and show damaging foliar symptoms if left uncontrolled. However, not much is known about the pathogenicity and virulence factors that *Pss* carries to cause disease in pepper seedlings. In this study, we investigated (i) the pathogenicity and virulence characteristics of *Pss* infecting pepper seedlings *in planta*, (ii) some of the major virulence factors of *Pss* strains *in vitro*, and (iii) the genetic content focusing on the core and variable virulence and antimicrobial genes carried by the *Pss* strains to better characterize and understand the virulence profile of *Pss* strains that infect pepper seedlings. We observed that the *Pss* strains differed in virulence (*Pss* bacterial population and disease symptoms) on pepper seedlings. With optimal disease conditions (28°C, 20%–80% relative humidity), disease symptoms appeared within 3 dpi with most *Pss* strains (*n* = 15/16) excluding SM226-1. The disease symptoms by most *Pss* strains (*n* = 15/16) increased rapidly (average number of lesions of 73.2 ± 54.3 at 3 dpi, 96.1 ± 68.0 at 7 dpi, and 245.5 ± 122.8 at 14 dpi), and after 14 dpi, diseased pepper seedlings were starting to lose leaves. This rapid and substantial disease progression of *Pss* strains on pepper seedlings further warrants more research to better understand the virulence of *Pss* in peppers so that it can be controlled. *In vitro* growth, biofilm, and motility assays were also conducted to understand the virulence of the *Pss* strains *in vitro*, and we found that strains differed in *in vitro* virulence. The *in vitro* variation in these phenotypes, however, did not correlate with virulence *in planta* between *Pss* strains.

Subsequently, whole-genome sequencing was utilized to study the genetic content of the *Pss* strains to further understand if genetic variation could influence variation in virulence among the 16 *Pss* strains. Phylogenetic studies using core SNPs indicated that all strains with the exception of SM914-13 were closely related. Nevertheless, we found a total of 812 genes in the variable genome (Fig. 5). The strains were clustered based on their variable gene profile to assess if strains with similar genetic profiles displayed similar virulence and pathogenicity traits. Some strains (SM207-3 and SM1038-14) with high virulence *in planta* also had a similar variable gene profile. Similarly, on the other end of the spectrum, two strains (SM226-1 and SM156-18) with the lowest virulence *in planta* also showed similar variable gene profiles. A multivariate correlation analysis and association analysis with Scoary were conducted to associate variable genes with *in planta* data to identify virulence genes that could help explain the observed variation in virulence of 16 *Pss* strains. However, after multiple-testing correction, no genes were significantly correlated, likely due to the small sample size of only 16 strains. In the future, as more *Pss* strains in pepper plants are isolated and sequenced, multivariate correlation analysis and Scoary need to be conducted with *in planta* data to identify virulence genes that correlate with *Pss*’s disease severity in pepper seedlings.

The genomic content analysis revealed the presence of several types of virulence genes in the *Pss* strains. Secretion system genes are known to play an important role in virulence and pathogenicity in phytopathogens like *Pss (27*). The *Pss* strains in this study contained a repertoire of secretion system genes (*n* = 94) that are reported to be associated with virulence in other *P. syringae* pathovars and some *Pss* strains in other host plants (27–29). Type III secretion systems are most commonly reported to be associated with virulence as T3SS is essential for virulence in phytopathogens like *Pss*. We found a total of 43 T3SS genes, of which 17 were part of the variable genome. The T3SS Hop effector genes were the most prevalent among the *Pss* strains variable genome. Many of these Hop effector genes are common among other *Pseudomonas syringae* pathovars including pathovar *tabaci*, *tomato* (DC3000), and *phaseolicola,* which are from different genomospecies that infect a wide range of host plants (30, 31). The Hop effector genes have a significant influence on the virulence of *Pss* strains as they modulate and weaken the host immune defense system pathways (30, 32).

Type VI secretion system (T6SS) genes were another set of secretion system genes that were present in the *Pss* genomes. We found a total of 18 T6SS genes, of which 4 were part of the variable genome. These genes were associated with the T6SS lipoprotein gene (*tssJ*), two PAAR repeat protein genes (*rhaS*), and a sigma-dependent regulator gene (*vasH*). Most *Pss* strains (*n* = 14/16) carried the *tssJ, rhaS,* and *vasH* genes. T6SS is part of a newly discovered secretion system that has been linked to virulence in phytopathogens (33). T6SS delivers toxic proteins into plant host cells and contributes to anti-inflammatory processes, virulence, and interbacterial competition (33). T6SS uses a tubular structure to deliver toxic proteins into the target cells. The *tssJ* (VasD) gene encodes for a core part of the tubular structure (34) whereas the *vasH* gene is a σ54-dependent transcriptional regulator of *hcp* genes that encode for a toxic protein called hemolytic co-regulating protein (Hcp) (33). The T6SS can sense environmental (host plant) or bacterial competition signals to activate the T6SS and release (via tubular structure-TssJ/VasD) toxic proteins like Hcp (regulated by the *vasH* gene) into the target cell. In other *P. syringae* pathovars, the deletion of any of these genes (*tssJ*/*vasD* or *vasH*) resulted in failure to assemble the T6SS or failure to release the Hcp protein, which subsequently resulted in reduction in virulence *in planta* (33–35). Similarly, these T6SS genes may be required for *Pss* to cause disease in pepper seedlings. Interestingly, the strains that caused low or no disease severity, SM156-18 and SM226-1, respectively, were missing these T6SS genes, while two of the strains that caused high disease severity, SM1038-14 and SM207-3, carried these T6SS genes.

Motility is an important virulence mechanism in *P. syringae*. In this study, we found a total of 87 motility genes, but only two motility genes (*pilA* and *flp*) associated with flagellar and twitching motility were part of the variable genome. The *pilA* gene has been reported to be important for surface motility in *P. syringae* pv. *tabaci* and subsequently for virulence as *pilA* mutants were unable to induce HR in host tobacco plants (36). Biofilm is another important virulence mechanism in phytopathogens, which helps them survive on the leaf surface and adhere to and colonize the apoplast and provides protection against antimicrobials (37). We found a total of 30 biofilm genes, but only two biofilm genes were part of the variable genome. These included genes that are part of the alginate biosynthesis gene cluster. Mutations in these genes within the alginate biosynthesis gene cluster in *Pss* infecting bean plants have been shown to reduce virulence and epiphytic fitness (38). Likewise, alginate biosynthesis genes like *algJ* could also play a role in the virulence and epiphytic fitness of *Pss* strains infecting pepper plants.

The whole-genome sequence analysis elucidated important antimicrobial resistance genes present in the 16 *Pss* strains. Almost half (*n* = 12/27) of the antimicrobial resistance genes found among the *Pss* strains were associated with copper resistance. All *Pss* strains carried genes associated with copper resistance, which is concerning because the use of copper-based antimicrobials is the most common chemical control method to manage phytopathogens like *Pss* in agriculture (14, 39). Our results provide further evidence for the lack of effectiveness of copper-based antimicrobials against phytopathogens and suggest a need for more strategic and comprehensive control methods to control phytopathogens like *Pss* in agriculture. The antimicrobial resistance profile of the *Pss* strains also indicated the presence of SAT for streptothricin resistance (closely related to streptomycin), beta-lactamase C genes associated with beta-lactam resistance, penicillin-binding protein for resistance to penicillin, DNA gyrase associated with fluoroquinolone resistance, and *nfxB* gene associated with quinolone resistance, and a multidrug resistance efflux gene (*cflA*) was also identified, which has been associated with resistance to various drugs including chloramphenicol. The presence of these antimicrobial resistance genes is additionally worrisome as these genes could spread to the environment (soil microbiome), animals, and consumers, creating a One Health issue.

Overall, this study provides useful insights into the pathogenicity and virulence characteristics of *Pss* strains *in vitro*, *in planta* in peppers, and their gene content. Exploring the gene content of *Pss* can be beneficial in understanding the potential virulence genes required to cause disease in pepper and in the future can help find drug targets to effectively manage *Pss* in peppers. In the future, more *Pss* strains in pepper need to be included to conduct *in silico*-based comparative multivariate correlation and association analyses to narrow down the list of virulence genes that might be associated with *Pss*’s ability to cause disease in peppers. Further, based on such a list, mutagenesis studies or gene expression studies *in planta* should be conducted to better facilitate the understanding of *Pss* genes essential for its virulence in pepper seedlings. Our findings provide an important first step toward understanding the virulence and antimicrobial gene content of *Pss* in peppers and represent the first study to explore the whole-genome sequences of *Pss* in peppers.

## MATERIALS AND METHODS

### Bacterial strains

Sixteen *Pss* strains collected from different counties in Ohio from *Pss* symptomatic pepper plants between 2013 and 2021 were used (Table S1). The *Pss* strains were grown in M9 minimal broth (composed of 33.7 mM Na_2_HPO_4_, 22 mM KH_2_PO_4_, 8.55 mM NaCl, 9.35 mM NH_4_Cl, 1 mM MgSO_4_, and 0.3 mM CaCl_2_, supplemented with 0.8% glucose), nutrient broth yeast (NBY) extract medium [nutrient broth (8 g), yeast extract (2 g), K_2_HPO_4_ (0.5 g), KH_2_PO_4_ (0.5 g), 1 M MgSO_4_ (1 mL), and 20% glucose (25 mL)], or LB broth at 28°C for 12–24 h in a shaking incubator at 180 rpm, unless indicated otherwise.

### Plant material

Bell pepper (*Capsicum annuum*) cultivar “California Wonder” seedlings were used for the *in planta* pathogenicity studies. California Wonder cultivars were used as they are bred to confer resistance to only tobacco mosaic virus and do not have resistance against *Pseudomonas syringae* strains. The seeds were sanitized using hot water (51°C) and chlorine (5.25%) treatment as described previously (40). Sanitized seeds were sown individually in 96-cell plug trays containing Baccto Professional Grower Mix (Baccto, Houston, TX) and grown in a greenhouse (with optimal growing conditions for pepper at 24°C–28°C temperature, 20%–80% relative humidity, and 12 h photoperiod) (41). The pepper seedlings were grown until the four-true leaf stage (ca., 6-week-old) prior to testing with 16 *Pss* strains. Plant experiments were conducted in a growth chamber with controlled temperature (24°C), relative humidity (80%), and photoperiod (12 h). Seedlings were watered daily in the pot.

### Collection, isolation, and identification of *Pss* isolates

Sixteen *Pss* strains isolated from infected pepper plants in northern Ohio (Wayne, Sandusky, and Seneca counties) from 2013 and 2021 were sourced from the OSU Miller lab collection (Table S1). Isolates were recovered from pepper seedlings showing round dark brown necrotic lesions around 2–3 mm with a yellow halo around them. Isolation and identification of the isolates were performed as previously described (15, 42). Standardized microbiological and biomolecular tests such as PCR and LOPAT (levan production, oxidase activity, pectolytic capability, arginine dihydrolase, and tobacco hypersensitivity) were conducted on the isolates suspected to be *Pseudomonas* to confirm that the strains belonged to LOPAT group Ia (details provided in Supplemental material), indicating that the strains are *P. syringae* strains (43). Further, species-specific PCR targeting *hrpZ* and *syrB* virulence genes (Table S7) was conducted to confirm the identification of *Pss* strains (18, 44, 45).

### *Pss* virulence on peppers

Six-week-old pepper seedlings (four-true leaf stage) were inoculated by spraying the foliage (5 cm away from the plant at a 45° angle) with a normalized *Pss* suspension (1 mL per seedling, 1.0 OD_600_ = approximately 1 × 10^9^ CFU per plant) with a commercial hand sprayer (Equate 8 oz plastic spray bottle, Texas, USA). The seedlings were rotated between every two sprays to ensure the entire seedling is sprayed. To ensure each seedling received equivalent *Pss* inoculum, an initial assessment was conducted where spraying 10 sprays from the 8 oz spray bottle resulted in the release of 1 g of liquid (equivalent to 1 mL) as measured using a weighing balance. This assessment was repeated multiple times (more than 10 times), and consistently, 1 g (1 mL) was obtained each time with 10 sprays. Therefore, we used this approach to spray 1 mL (10 sprays using 8 oz spray bottle) on each seedling when inoculating the seedlings. The *Pss* strain inoculum was prepared as described above in M9 minimal broth at 28°C at 180 rpm overnight. Inoculated seedlings were incubated in a growth chamber (28°C, 20%–80% relative humidity, 12 h photoperiod). The seedlings (*n* = 14 per treatment group) were watered daily in the pots. Starter fertilizer [2 lbs/50 gal of 12–48–8 (%) nitrogen–phosphorus–potassium (N-P-K)] was applied to the seedling in the greenhouse. The disease severity (number of lesions on the surface of two lower leaves for each seedling) was recorded at 3, 7, and 14 dpi. The disease incidence (number of seedlings showing PLS symptoms) was recorded at 3, 7, and 14 dpi. The bacterial load in inoculated seedlings was determined at 0, 3, 7, and 14 dpi using the direct dilution plating method (46). Briefly, seedlings (1 cm above the soil) were collected individually in Whirl-Pak bags. The weight of each seedling was recorded prior to adding 2 mL of 1× phosphate buffered saline (PBS). The seedlings were macerated, and the macerate was serially diluted and plated on an NBY agar medium supplemented with copper sulfate (25 µg/mL). NBY plates were supplemented with a non-lethal dose of copper sulfate to create a semi-selective growing condition to reduce the background growth coming from the phytobiome. Colonies characteristic of *Pss* were counted after 48-h incubation at 28°C. The experiment was performed twice with 14 biological replicates per strain in each experiment, and plants treated with PBS were used as a negative control.

### Bacterial growth assay

The growth of *Pss* in M9 minimal broth was assessed in nutrient-limiting conditions mimicking the limited nutrient conditions present *in planta*. Each *Pss* strain was grown for 12 h in M9 minimal broth at 28°C at 180 rpm until reaching the exponential phase. The OD_600_ was adjusted to 0.05 (approximately 1 × 10^7^ CFU/mL) in fresh M9 minimal broth. In a sterile, non-treated, flat-bottom 96-well plate (Corning Inc, Corning, NY, USA), 100 µL of the adjusted bacterial culture was transferred per well (*n* = 3 reps per strain). The plate was incubated at 28°C for 48 h in a Sunrise Tecan kinetic microplate reader (San Jose, CA, USA), and kinetic measurements were conducted at OD_600_ every 15 mins. The experiment was conducted twice with M9 minimal broth as a negative control. The turbidimetric data were used to determine the growth rate per hour and doubling time, which was calculated using the growthcurver 0.3.1 package in R 4.3.1.

### Biofilm assay

The production of biofilm by the *Pss* strains was assessed using the crystal violet method (47). Each *Pss* strain was grown for 12 h in M9 minimal broth at 28°C at 180 rpm, when they reached the exponential growth phase. The OD_600_ was adjusted to 0.05 (approximately 1 × 10^7^ CFU/mL) in fresh M9 minimal broth. In a 96-well plate, 100 µL of the adjusted bacterial culture was transferred per well (*n* = 3 reps per strain). The plate was incubated for 72 h at 28°C without shaking; then, the supernatant was carefully removed, and the wells were gently washed by adding 175 µL sterile water to remove any planktonic bacteria. The water was discarded, and the biofilm was stained by adding 175 µL of 0.1% crystal violet solution to each well for 15 mins (47). The plate was washed three times using 200 µL of sterile water as described above to remove excess crystal violet. The plate was dried at room temperature before the stained biofilm was solubilized in 200 µL of 95% ethanol. An absorbance microplate reader was used to measure the OD_570_ to quantify the level of biofilm in each well. The experiment was conducted twice. Wells treated with M9 minimal broth were used as a negative control.

### Motility assay

A motility assay was conducted on the *Pss* strains on semi-solid agar (47). Each *Pss* strain was grown for 12 h in LB broth at 28°C at 180 rpm. The OD_600_ was adjusted to 0.05 (approximately 1 × 10^7^ CFU/mL) in fresh LB broth. One milliliter of 0.3% semi-solid LB agar supplemented with 0.01% tetrazolium chloride was poured into each well of a 24-well plate. A 0.3% agar concentration was used as swarming motility can be observed at this concentration (48). A 31-gauge, 8-mm sterile syringe needle was used to transfer approx. 1 µL of the adjusted bacterial culture into the semi-solid agar at the center of the well using a stabbing motion (*n* = 2 replicates per strain). The plate was sealed using parafilm to avoid evaporation of the water in the agar. The plate was incubated at 28°C without shaking for 24 h. Wells that received LB were negative controls. The motility of each of the strains was quantified by measuring the diameter of the halo zone after 24 h of incubation. The experiment was conducted twice.

### Genomic DNA extraction

Genomic DNA was extracted using a Wizard Genomic DNA Purification Kit (Promega, Madison, WI, USA), following the manufacturer’s instructions. *Pss* strains were grown in fresh NBY agar plates overnight at 28°C. A loopful of bacterial colonies were collected from the NBY plates and resuspended in 1-mL sterile water. The bacterial suspensions were vortexed thoroughly and then centrifuged for 7 mins at 4,500 rpm using a benchtop centrifuge. The supernatant was discarded, and Nuclei Lysis solution was added to lyse the bacterial cells to release genomic content. RNase solution and Protein Precipitation solution were added to extract and remove any RNA and proteins from the bacterial cells. The remaining DNA was washed using 70% ethanol and precipitated with absolute alcohol to remove any remaining protein and salt traces (49). The DNA was resuspended in 100 µL nuclease-free water. DNA quality and quantity were validated using the NanoDrop Microvolume Spectrophotometer (Thermo Scientific, Waltham, MA, USA). DNA samples with a 260/280 ratio of approx. 1.8 and a 260/230 ratio of 2.0–2.2 were considered standards for pure DNA samples.

### Whole-genome sequencing of *Pss* pepper isolates

Genomic DNA was extracted as described, and only genomic DNA with high quality and quantity (concentration above 50 ng/µL) was used for sequencing (50). A genomic DNA Clean and Concentrator kit (Zymo Research, Irvine, CA, USA) was used to ensure the quality of extracted DNA. Whole-genome sequencing was performed on the Illumina Miseq platform using V2 chemistry with 2 × 250 paired-end chemistry (51). The Nextera XT DNA Sample Prep Kit (Illumina Inc., San Diego, CA, USA) was used to prepare a genomic DNA library (0.3 ng/µL) for sequencing, following the manufacturer’s instructions. The libraries were normalized using a bead-based procedure and pooled together at equal volumes. The pooled library was denatured and sequenced using Miseq reagent version 2, as described previously (Illumina Inc.) (51, 52).

The quality of the reads in the raw FASTQ files was checked using FastQC (Babraham Bioinformatics, Cambridge, MA, USA) as previously described (53, 54). Trimmomatic (v.0.36) (55) was used to remove adapters and bad-quality reads; reads with a Phred quality score below 20 were trimmed. The cleaned paired-end reads were *de novo*-assembled using SPAdes (v.3.6.0) (56) with specified k-mer sizes of 21, 33, 55, 77, 99, and 127 mer length (52). The quality of the resulting assemblies was assessed using QUAST (v.5.0.2) (57) and CheckM (v.1.1.2) (58): QUAST was used to determine the number of contigs, genome size, and GC content, while CheckM was used to determine genome completeness and contamination using lineage-specific markers (57, 58). The average depth of coverage of each contig was computed by mapping the reads to the assembled contigs using BBmap (v.35.49) (59). The pangenome and core genome rarefaction curves were generated using the ggcurves function from the R package pagoo (v.0.3.18) (60). Core genes (genes present in at least 99% of genomes), soft core genes (genes present in 95%–99% of genomes), shell genes (genes present in 15%–95% of genome), and cloud genes (genes present in <15% of genomes) were identified using Roary (v.3.11.2) (21). Heaps’ law was applied to determine the openness of the pangenome using micropan package in R; the pangenome was considered to be closed if the alpha value was >1 (61).

### Phylogenomic analysis

To determine the phylogenetic relationship between the 16 *Pss* strains from this study and available reference genomes of *P. syringae* pathovars, kSNP3 (v.3.1) was used. kSNP3 is a reference-free and alignment-free method used to identify sites with SNPs that are shared among the assembled genomes (62). kSNP3 uses maximum likelihood approaches to build a phylogenetic tree (63). Twelve different pathovars of *P. syringae* (7 *Pss* and 11 non-*Pss* strains closely related to *Pss*) were downloaded from NCBI’s GenBank to further validate that the strains collected in this study are part of the pathovar *syringae* (Fig. 3; Table S3). The phylogenetic tree was rooted using *Pseudomonas moraviensis* as the outgroup taxa. kSNP3 first identifies core SNPs, i.e., those present in all input genomes (*n* = 34, 16 *Pss* strains from this study, 7 *Pss* strains from NCBI, and 11 *P*. *syringae* strains from different pathovars). For visualization of the phylogenetic tree, ggtree was used in R (64).

### Annotation of gene content including virulence, antimicrobial resistance, plasmid, and prophage genes

The assembled genomes were annotated and analyzed using open-source analytical tools and pipelines [SEED (http://pubseed.theseed.org/) and RAST (v.1.073)] (23). RAST was used to annotate *Pss* genes, and SEED clustered the genes into specific subsystems (52). Annotated genomes of *Pss* strains were compared against four different antimicrobial resistance gene databases (ResFinder, CARD, NCBI, and ARG-ANNOT) (65) using ABRicate (v.1.0.1) (https://github.com/tseemann/abricate) (66). Similarly, ABRicate was used to identify virulence genes in our *Pss* strains by comparing them against the VFDB (67). BastionHub, T3SEdb, *Pseudomonas* genome database (https://pseudomonas.com/), and a Hop database for T3SS of *P. syringae* were also used to identify different secretion system genes in our *Pss* strains (68–70). Customized databases were made in ABRicate by downloading the fasta sequences from the BastionHub database (https://bastionhub.erc.monash.edu/download.jsp) and T3DB. The AA fasta sequences obtained from the BastionHub database were converted to nucleotide sequences using EMBOSS Backtranseq. PHASTER (https://phaster.ca/) was used to identify the prophage population in the *Pss* genomes (52). MOB-Suite (v.3.1.0 ) (71) was used to identify the plasmids in the *Pss* genome (71).

### Gene and SNP association analysis

Annotated *Pss* genomes were used to separate the core and variable genomes. The core and variable genomes were defined based on the presence of the gene in all strains or absence of the gene in at least one strain, respectively. Hierarchical clustering analyses and principal component analyses were used to identify differences in the variable genome (gene content) between the strains. Further, principal component and multivariate correlation analyses were used to compare the variable genomes *in vitro* (growth, biofilm, and motility) and *in planta* (disease severity at 3 and 7 dpi) to identify genes associated with specific phenotypic characteristics. A BH false discovery rate (FDR) correction for multiple testing was applied with an FDR-adjusted *P* < 0.05 for the multivariate correlation analysis between the variable genomes with *in vitro* (growth, biofilm, and motility) and *in planta* (disease severity at 3 and 7 dpi) characteristics (72). The multiple-testing-corrected *P*-values were obtained by using p.adjust function from the stats 4.3.1 package in R 4.3.1. A linear regression analysis was performed between the disease severity of 3 and 7 dpi with independent variables such as growth, motility, and biofilm in bacteria in R 4.3.1 using the stats 4.3.1 package at a significance level of *P* < 0.05.

Additionally, Scoary v1.6.16 was also used to identify genes within the variable genome that were significantly associated with *in vitro* (motility, biofilm formation, bacterial growth) and *in planta* (disease severity at 3 and 7 dpi) characteristics (21). A gene presence–absence file generated by Roary was used as the genotypic data, while motility, biofilm-producing ability, bacterial growth, and disease severity were used as the phenotypic data. An arbitrary threshold was set for each *in vitro* and *in planta* characteristic (66). For the motility data set, strains with >5.3 mm diameter were considered highly motile, OD_570_ > 0.38 was considered high biofilm producers, and OD_600_ > 0.65 was considered fast growers. Disease severity indices (number of lesions) greater than 64 and 80 on 3 and 7dpi, respectively, were considered to have high disease severity. Genes with a Benjamini–Hochberg-corrected *P*-value <0.05 were considered significant (72).

To distinguish between functional and non-functional gene copies, Pseudofinder (v.1.1.0) (was used to screen for candidate pseudogenes in each assembled genome, using *Pseudomonas syringae* ASM14582v2 as the reference genome (73). Pseudofinder was also provided with a DIAMOND (74)(database created from the NCBI Non-Redundant protein database, which was downloaded on 24 December 2022. The genomic coordinates of pseudogenes identified by Pseudofinder were intersected with those of the list of identified virulence genes (motility, biofilm, and secretion system) using the “bedtools intersect” command from Bedtools (v.2.30.0) (71). Next, the table of virulence gene copy numbers in each assembly was modified to contain functional gene copy numbers only: pseudogenic copies of a gene were subtracted from the total copy number of that gene. A multivariate correlation analysis was conducted with the *in planta* (disease severity) data to identify genes whose functional copy number is associated with specific phenotypic characteristics, using thresholds of *r*^2^ > ±0.5 and *P* < 0.05.

To identify SNPs for a SNP-based association analysis, the “snippy-multi” command from Snippy (v.4.6.0) (https://github.com/tseemann/snippy) was run with default settings, and SM914-13 (the most complete and high-quality assembly in our study) was used as the reference genome (75). Snippy used FreeBayes v.1.3.6 to call and SnpEff v.5.0 to annotate SNPs. SNPs annotated as “synonymous_variant,” “intergenic_region,” “intragenic_variant,” and “intron_variant” were removed in order to only select non-synonymous SNPs with potential functional consequences (76). Similar to the pseudogene analysis, SNPs in virulence genes were selected by intersecting the genomic coordinates for the non-synonymous SNPs with those of the virulence genes using “bedtools intersect,” tabulated SNPs per gene per isolate, and a multivariate coordinate analysis with the *in planta* (disease severity at 3 and 7 dpi) data was conducted to identify SNPs associated with specific phenotypic characteristics. A BH FDR correction for multiple testing was applied with an FDR-adjusted *P* < 0.05 for the multivariate correlation analysis between the identified SNPs and *in planta* (disease severity at 3 and 7 dpi) characteristics (72). The multiple-testing-corrected *P*-values were obtained by using p.adjust function from the stats 4.3.1 package in R 4.3.1.

### Statistical analysis

All statistical analysis on the experimental data was conducted using JMP Pro17 (SAS, Cary, NC, USA). Continuous data sets for *in vitro* and *in planta* experiments were tested for normality and variance using the Shapiro–Wilk test prior to performing a parametric one-way ANOVA combined with a Tukey test (*P* < 0.05). Several tests were also conducted to test for equal variance between the two trials conducted for each of the *in planta* experiments. Five tests were conducted to test for equal variance between trials, including the O’Brien test, Brown–Forsythe test, Levene test, Bartlett test, and two-sided *F*-test (*P* < 0.05). Randomization of the groups within the growth chamber was conducted for each plant experiment. Bacterial load and disease severity data were log-transformed before performing statistical analyses.

## Data Availability

All sequencing data used in this study are available in the NCBI Sequence Read Archive under the project number BioProject ID PRJNA1035930.

## SUPPLEMENTAL MATERIAL

Supplemental figures spectrum.00064-24-s0001.pdf Fig. S1-S8.

Supplemental material spectrum.00064-24-s0002.docx Description.

Table S1 spectrum.00064-24-s0003.docx Year and location of isolation from peppers, LOPAT and PCR results.

Table S2 spectrum.00064-24-s0004.xlsx Correlated genes detected by the multivariate correlation analysis and Scoary.

Table S3 spectrum.00064-24-s0005.xlsx Pseudomonas syringae strains (n=18) both within and outside the P. syringae pv. syringae phylogenetic group.

Table S4 spectrum.00064-24-s0006.xlsx Variable genes (n=812) within the 16 Pseudomonas syringae pv. syringae (Pss) strains.

Table S5 spectrum.00064-24-s0007.xlsx Multiple testing correction for all the variable genes (n=142 and 151).

Table S6 spectrum.00064-24-s0008.xlsx Pseudogenes (n=184) present in the variable genome of the 16 Pseudomonas syringae pv. syringae (Pss) strains.

Table S7 spectrum.00064-24-s0009.xlsx Multiple testing correction for SNPs.

Table S8 spectrum.00064-24-s0010.docx Pathovar-specific primers used in PCR assays to identify Pseudomonas syringae pv. syringae (Pss) strains from peppers.

ASM does not own the copyrights to Supplemental Material that may be linked to, or accessed through, an article. The authors have granted ASM a non-exclusive, world-wide license to publish the Supplemental Material files. Please contact the corresponding author directly for reuse.
