# Supplementary figures and images for: In vitro, in planta, and comparative genomic analyses of Pseudomonas syringae pv. syringae strains of pepper (Capsicum annuum var. annuum)
Source: Microbiol Spectr. 2024 May 7;12(6):e00064-24. doi: 10.1128/spectrum.00064-24 (PMC11237606; doi:10.1128/spectrum.00064-24)

Fig. S1

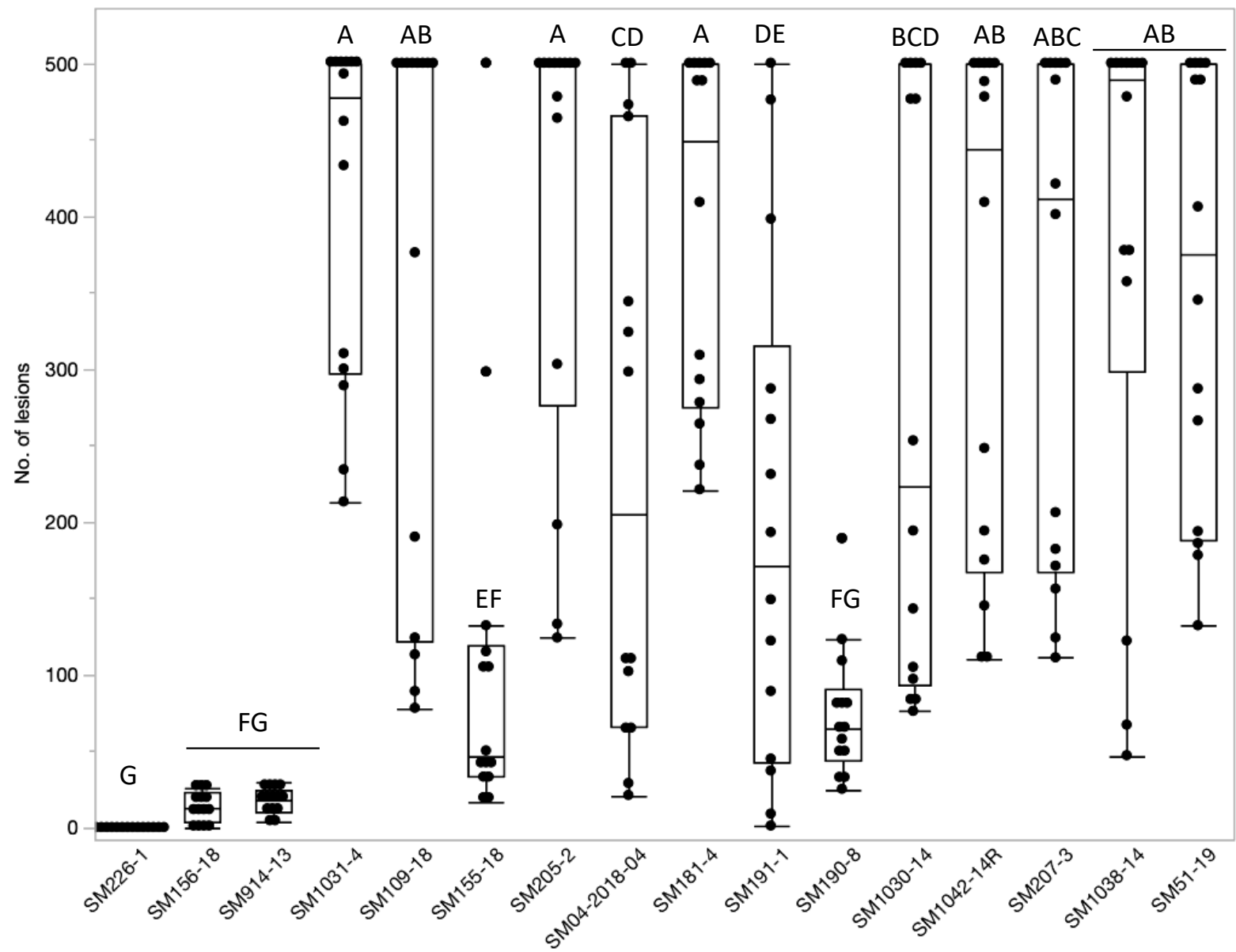

Fig. S2

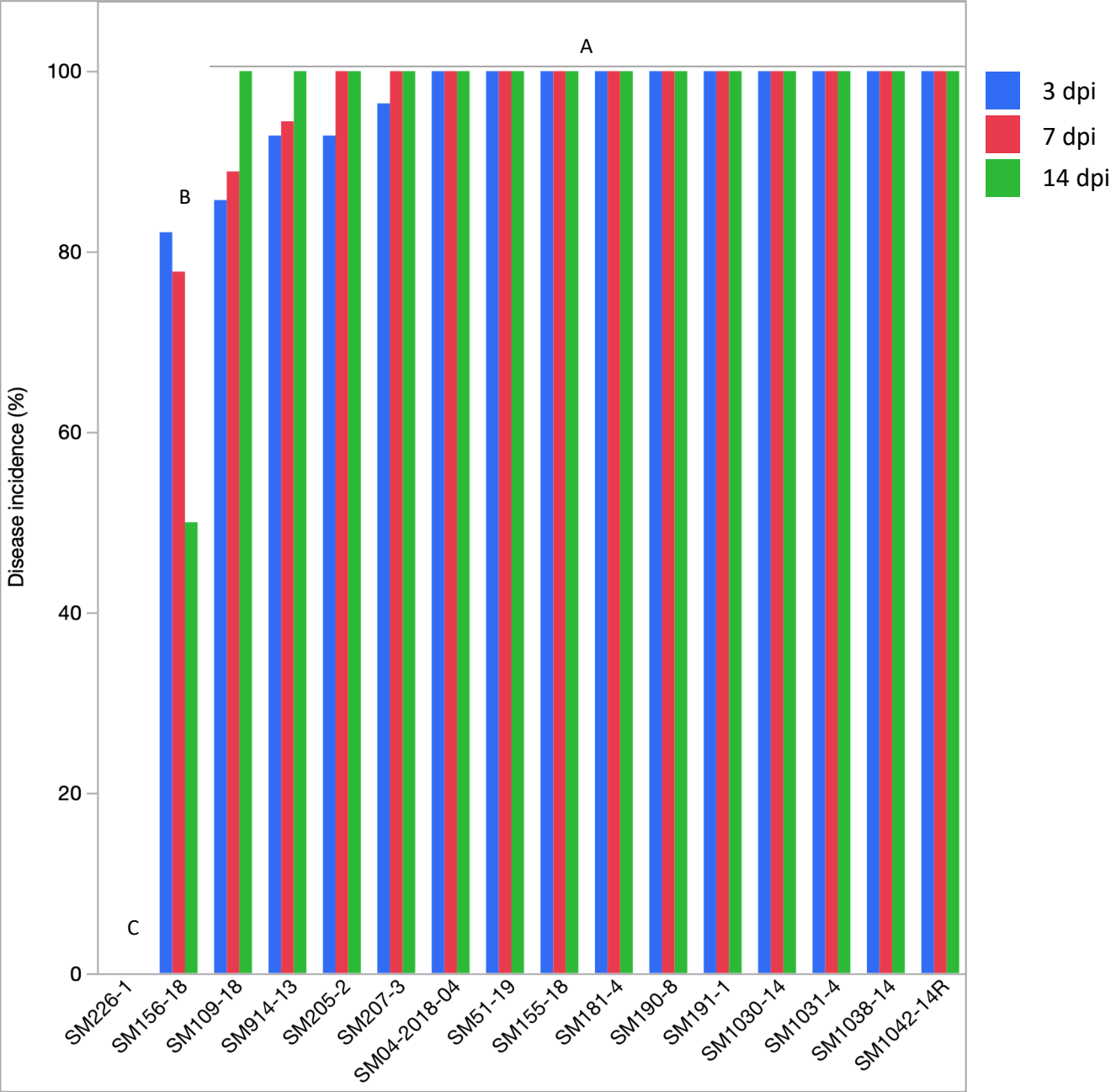

Fig. S3

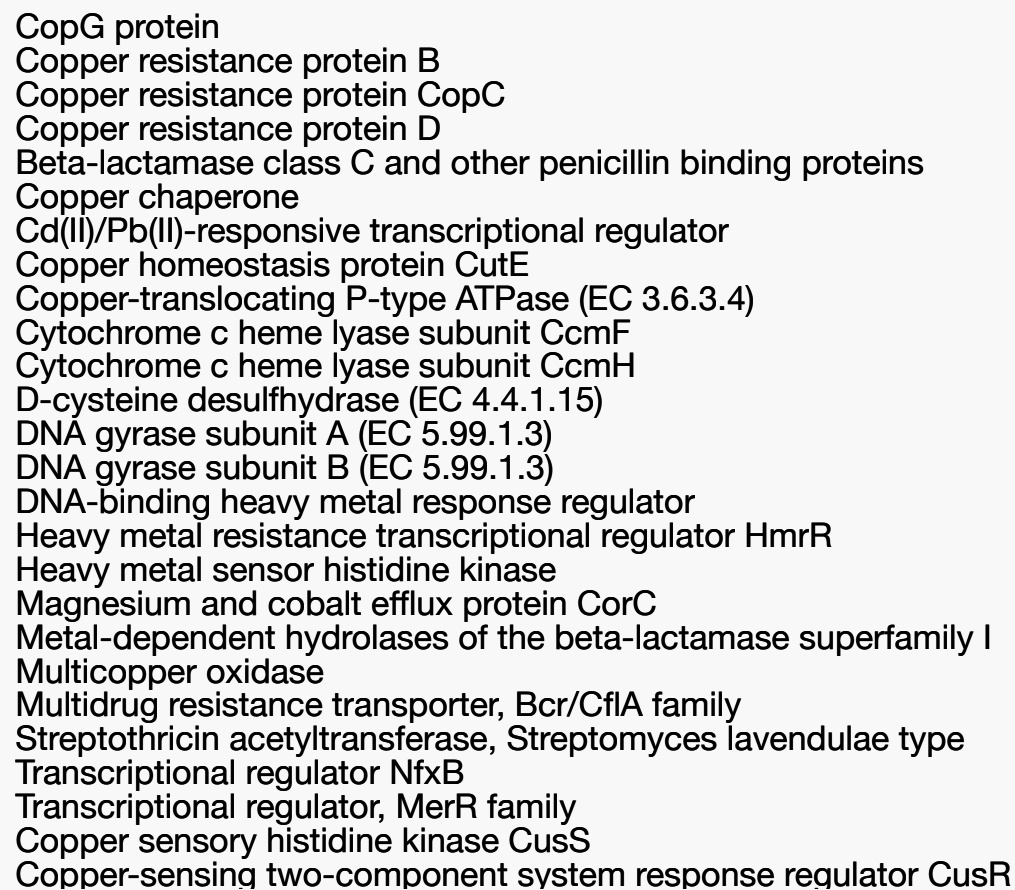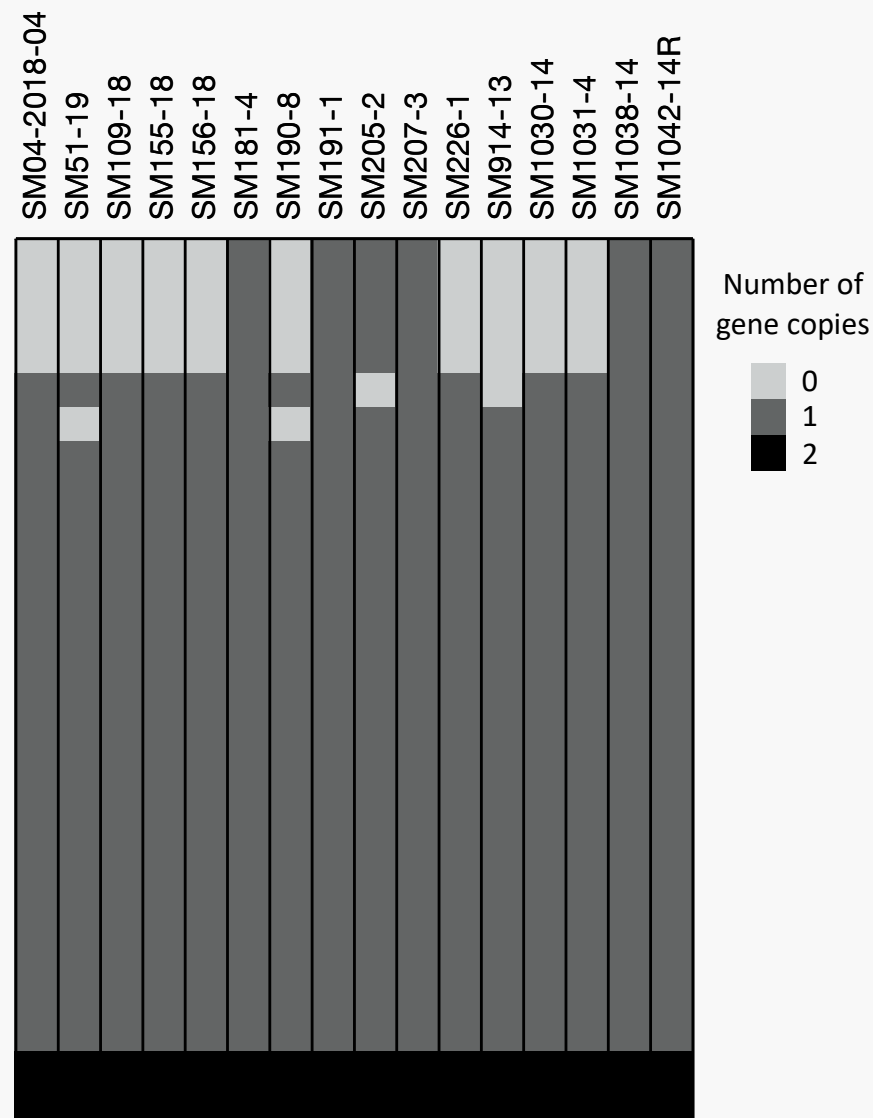

Fig. S4

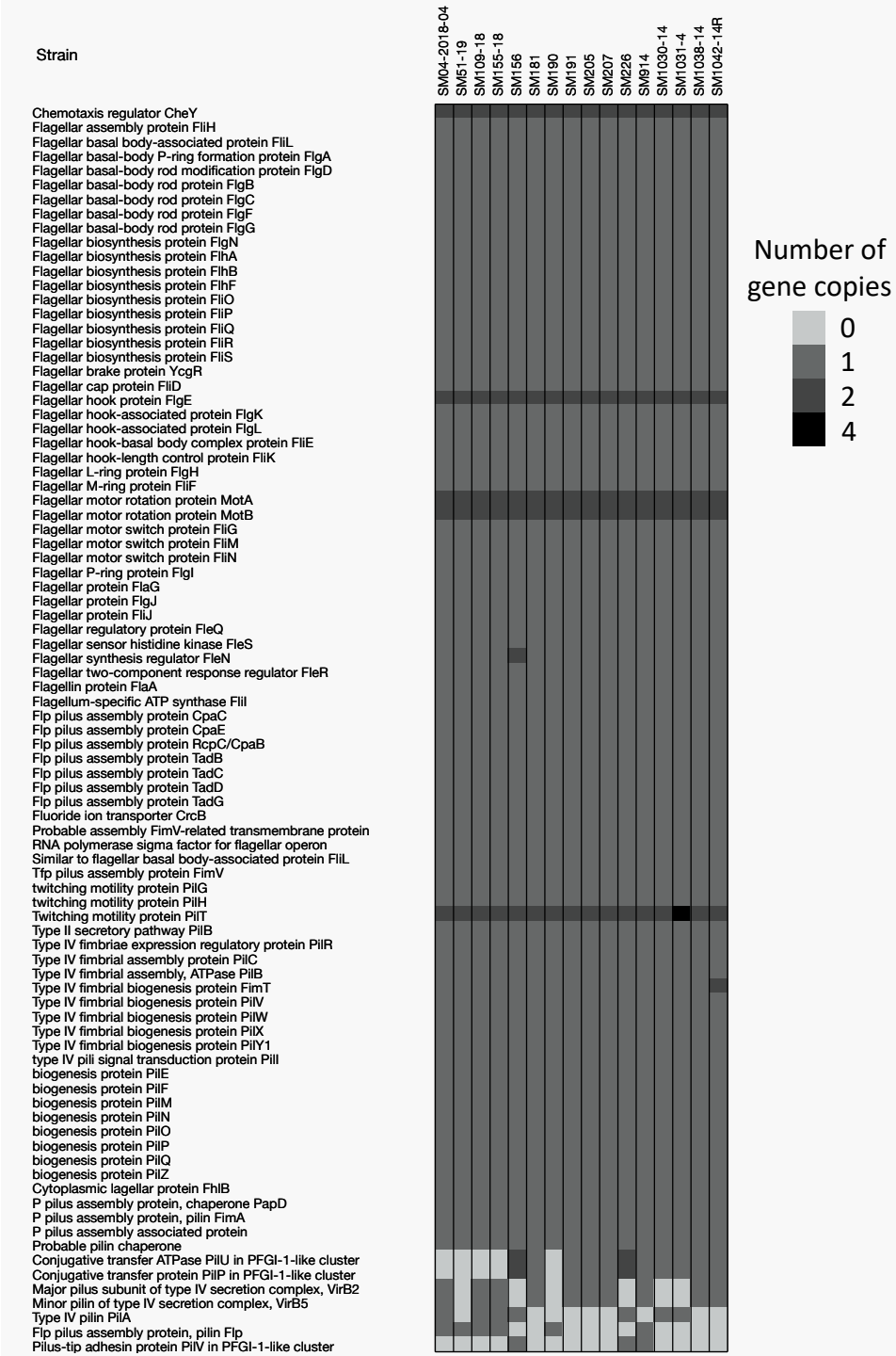

Fig. S5

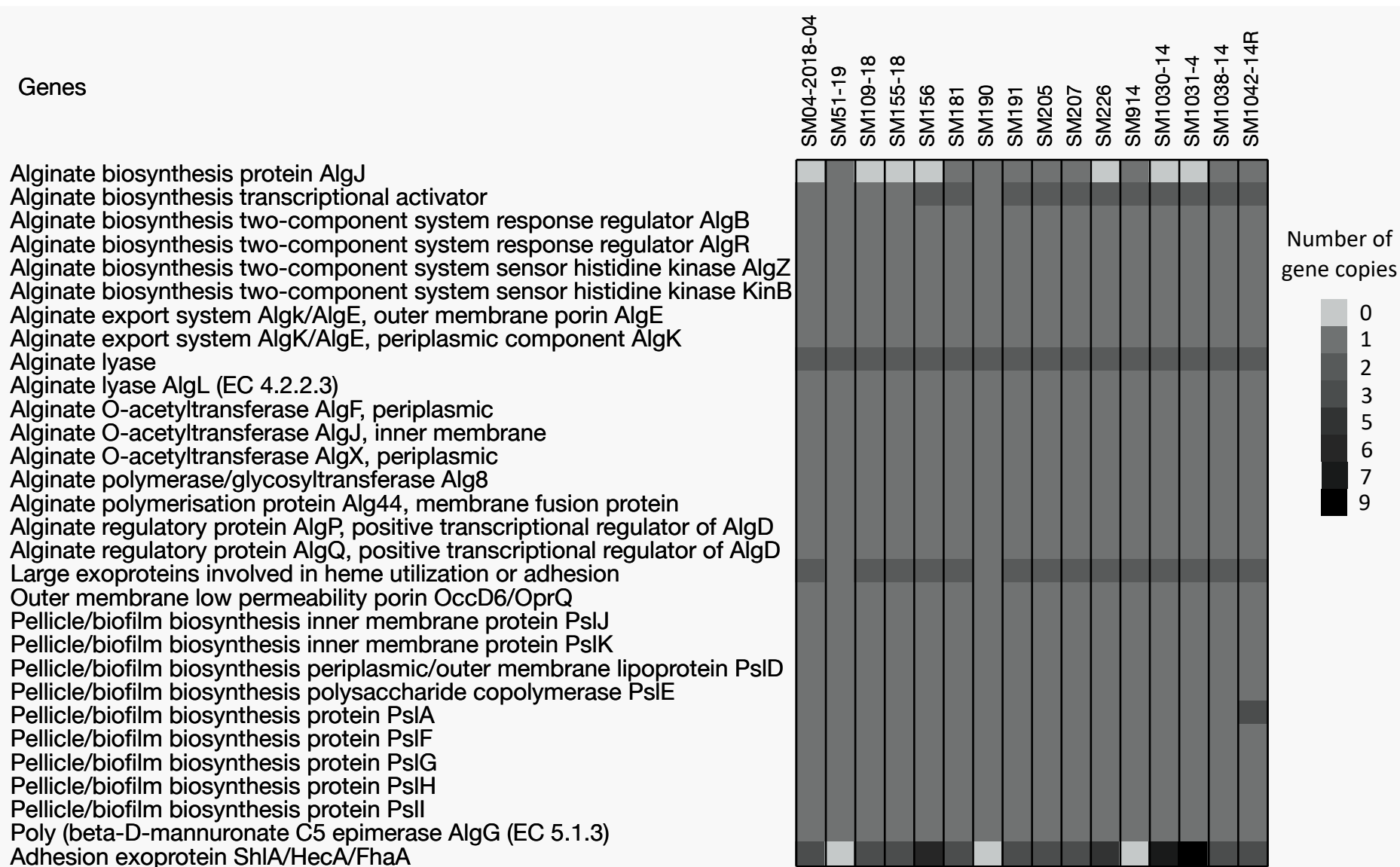

Fig. S6

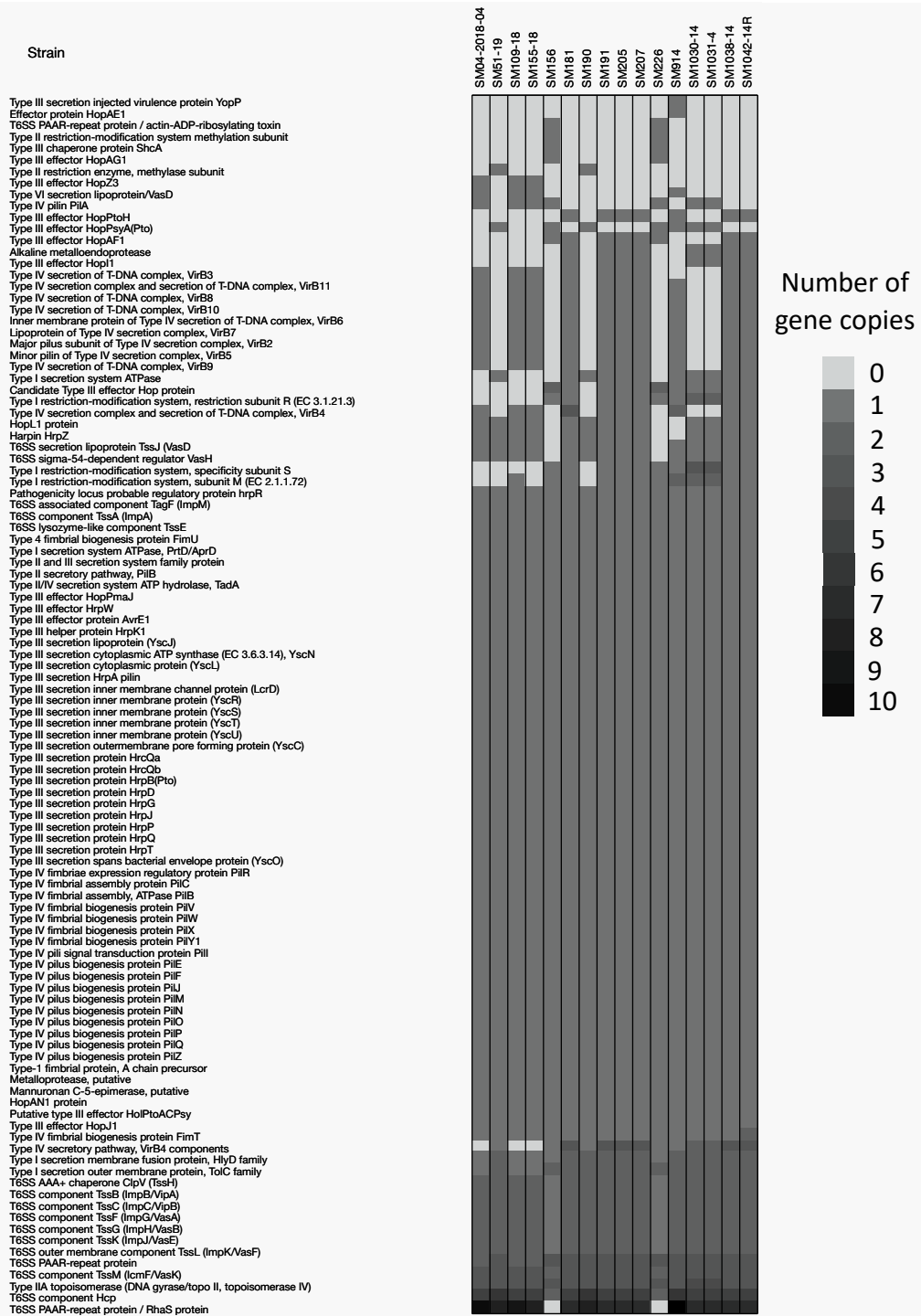

Fig. S7

## Correlated genes

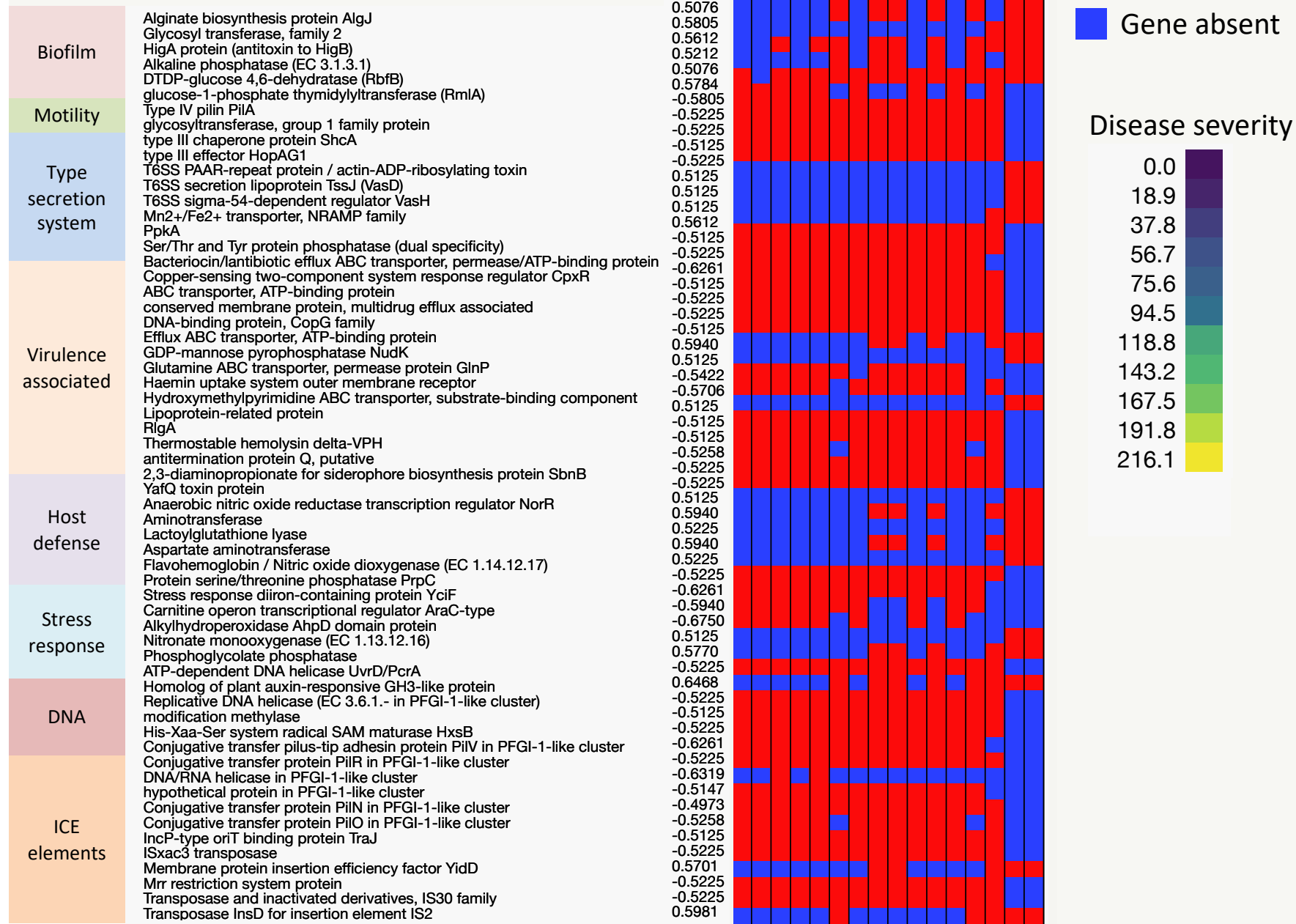

Fig. S8

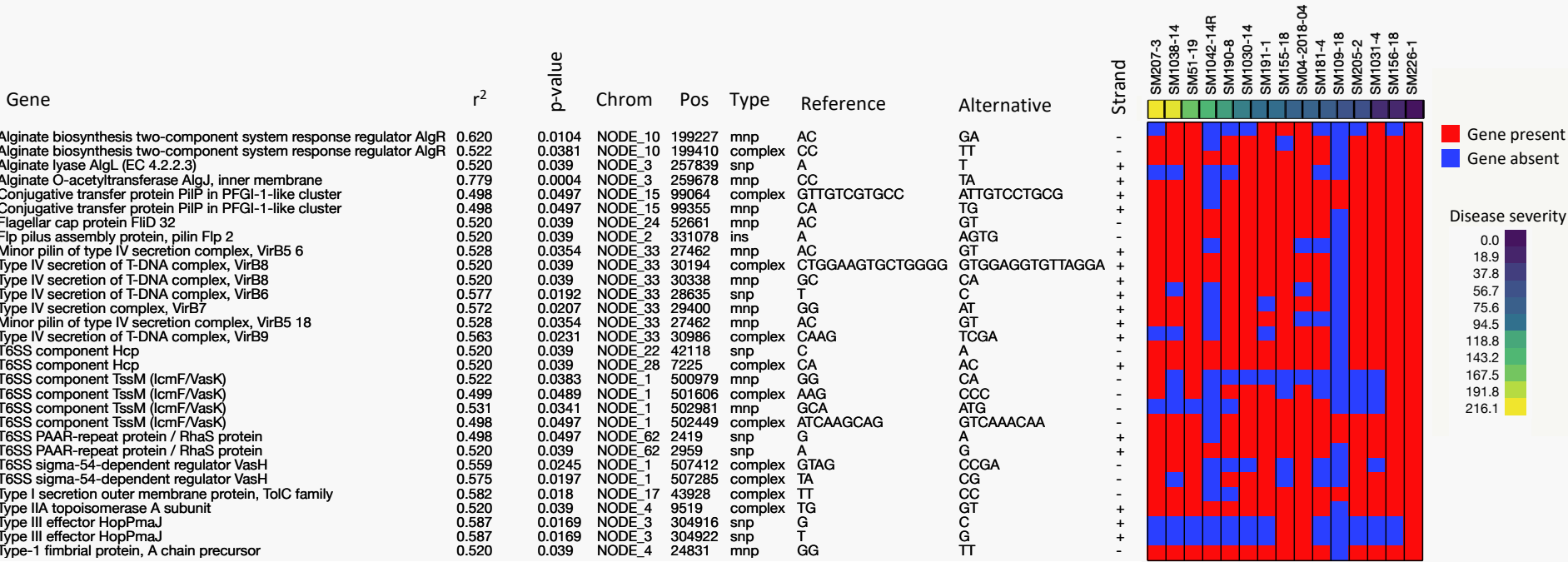

Supplement: Supplemental figures — Fig. S1-S8. [file spectrum.00064-24-s0001.pdf]
