# Supplementary material for: In vitro, in planta, and comparative genomic analyses of Pseudomonas syringae pv. syringae strains of pepper (Capsicum annuum var. annuum)
Source: Microbiol Spectr. 2024 May 7;12(6):e00064-24. doi: 10.1128/spectrum.00064-24 (PMC11237606; doi:10.1128/spectrum.00064-24)
Supplement: Supplemental material — Description. [file spectrum.00064-24-s0002.docx]

**Supplemental material:**

**Identification of Pss strains using PCR.**Pathovar-specific PCR targeting two genes (*hrpZ* and *syrB*) was conducted to identify the pathovar of the isolates. A set of primers amplifying the gene coding for a virulence protein factor (*hrpZ*), which is a part of the hrp gene cluster (hypersensitivity repones and pathogenicity), was used (1, 2). Another set of primers that amplifies the gene encoding syringomycin production (*syrB*), a phytotoxin produced by P. syringae pv. syringae, was also used (3, 4). The PCR mix was made with 2 µl of DNA with 100 ng of genomic DNA, 10 pmol of each primer set, GoTaq Green Master (Promega) Mix (containing Taq DNA polymerase, dNTPs, MgCl_2_, and reaction buffers), and nuclease-free water. The PCR amplification was conducted using the Eppendorf EP Master Cycler S machine. The initial denaturation was set for 94˚C for 5 mins, followed by 35 cycles of denaturation at 94˚C for 30 s each, annealing for 30 s at designated temperature (**Table S2**), extension at 72˚C for 30 s, and final extension at 72˚C for 10 mins (1, 4). PCR products (5µl) were visualized under UV light using a 1% agarose gel after running electrophoresis for 30 mins at 100 V. Water and DC3000, a known P. syringae pv. tomato strain distinct from Pss strains in their genetic and pathogenicity profile were used as the control in both PCR tests.

**Characterization of Pss strains using LOPAT**. The LOPAT test consists of five biochemical tests for the identification of Pseudomonas *syringe* (5). The tests included levan production, oxidase reaction, arginine dihydrolase activity, and hypersensitive reaction (HR) on tobacco leaves (6). The production of levan was validated by plating the Pseudomonas isolates on nutrient agar medium supplemented with 5% sucrose (6). The plates were incubated for 3-5 days at 28˚C. All 16 Pseudomonas isolates showed presence of large, white-domed mucoid colonies indicative of levan production (7). Because P. syringae strains are known to be levan producers, the isolates producing levan on nutrient agar are likely to be P. syringae strains.

For the oxidase tests, commercial OxiStrips (Hardy Diagnostics, Springboro, OH, USA) containing 1% N, N, N′, N′-Tetramethyl-p-phenylenediamine dihydrochloride (TMPD) solution were used (6). Freshly grown inoculum of the Pseudomonas isolates was streaked onto the strip after moistening the strip with sterile water. The isolate is considered positive for oxidase if it turns dark purple within 15s. A positive oxidase activity indicates the presence of cytochrome oxidase (8). *P.syringae* are generally negative for oxidase. The 16 Pseudomonas isolates in this study did not turn purple in color during the oxidase test indicating that they are negative for oxidase activity.

The production of pectolytic enzymes was verified by pipetting 10 µl of bacterial suspension of the Pseudomonas isolates (10^8^ CFU/ml) on alcohol-flamed potato slices (7-8 mm thickness) (6). The inoculated potato slices were placed in a Petri dish containing moistened sterile filter paper and incubated at 22˚C in a moist chamber. After 24 h., if soft rot symptoms are observed then the isolates possess pectolytic enzymes. P. syringae generally does not contain pectolytic enzymes and should test negative for pectolytic activity in potatoes (6). The 16 Pseudomonas isolates in this study did not show soft rot symptoms after 24 h indicating that they are negative for pectolytic activity in potatoes.

Arginine dihydrolase activity tests for the presence of two enzymes that allows pseudomonads to grow under anaerobic conditions. To determine the presence of arginine dihydrolase in the isolates, a fresh culture of Pseudomonas isolates was stabbed onto a soft agar tube (4 ml) of Thornley’s medium 2A (Peptone (1 g), NaCl (5 g), K_2_HPO_4_ (0.3 g), Agar (3 g), Phenol red (0.01 g), Arginine HCl (10 g), pH 7.2) (6).The tube was sealed with 1 ml of sterile mineral oil and incubated at 28˚C for 4 days. A change in color to pink within 4 days indicates the presence of arginine dihydrolase. *P. syringae* isolates should not show any color change as they are negative for arginine dihydrolase. The 16 Pseudomonas isolates in this study did not change in color to pink after 4 days, indicating that they are for arginine dihydrolase.

A tobacco hypersensitive reaction (HR) is used to determine pathogenic pseudomonads. Pseudomonas strains that cause local cell death have an HR in tobacco plants. Strains that cause HR in tobacco plants are considered pathogenic. The abilitycapability ofthe 16 Pseudomonas isolates from this study to induce hypersensitivity (HR) was tested with ‘Samsun’ tobacco seedlings (6). The isolates were grown overnight in NBY broth at 28˚C in a shaking incubator. The bacterial cultures were normalized to 0.001 OD_600_ (approximately 1x10^6^ CFU/ml) in 1 X PBS (Phosphate Buffered Saline). Tobacco leaves were inoculated with the normalized bacterial culture by the leaf infiltration method using sterile syringes (9). A positive HR response is indicated by the presence of chlorotic and necrotic symptoms (indicators of HR) at the inoculation site 24 h post-inoculation (10). The 16 Pseudomonas isolates showed a presence of chlorotic and necrotic symptoms, indicating that they have positive HR response. P. syringae strains generally are positive for HR response in tobacco plants as P. syringae are pathogenic bacteria. P. syringae pv. tomato DC3000 was used as a control that is positive for HR response on tobacco plants*.* Seedlings treated with PBS was used as negative control (negative HR in tobacco).

**References:**

1. Yoshioka R, Uematsu H, Takikawa Y, Kajihara H, Inoue Y. 2020. PCR detection of *Pseudomonas syringae* pv. *syringae*, the causal agent of bacterial black node in barley and wheat, using newly designed primer sets. J Gen Plant Pathol 86:387–392.

2. Charkowski AO, Huang HC, Collmer A. 1997. Altered localization of HrpZ in *Pseudomonas syringae* pv. *syringae* hrp mutants suggests that different components of the type III secretion pathway control protein translocation across the inner and outer membranes of gram-negative bacteria. J Bacteriol 179:3866–3874.

3. Peng L, Yang S, Zhang Y, Haseeb Y, Song S, Xu X, Yang M, Zhang J. 2022. Characterization and Genetic Diversity of Pseudomonas syringae pv. *syringae* Isolates Associated with Rice Bacterial Leaf Spot in Heilongjiang, China. 5. Biology 11:720.

4. Sorensen KN, Kim K-H, Takemoto JY. 1998. PCR Detection of Cyclic Lipodepsinonapeptide-Producing *Pseudomonas syringae* pv. *syringae* and Similarity of Strains. Applied and Environmental Microbiology 64:226–230.

5. Nikolić I, Stanković S, Dimkić I, Berić T, Stojšin V, Janse J, Popović T. 2018. Genetic diversity and pathogenicity of *Pseudomonas syringae* pv. *aptata* isolated from sugar beet. Plant Pathology 67:1194–1207.

6. Schaad NW, Jones JB, Chun W. 2001. Laboratory guide for the identification of plant pathogenic bacteria. Laboratory guide for the identification of plant pathogenic bacteria.

7. Lelliott RA, Billing E, Hayward AC. 1966. A Determinative Scheme for the Fluorescent Plant Pathogenic Pseudomonads. Journal of Applied Bacteriology 29:470–489.

8. Kovacs N. 1956. Identification of *Pseudomonas pyocyanea* by the Oxidase Reaction. 4535. Nature 178:703–703.

9. Chincinska IA. 2021. Leaf infiltration in plant science: old method, new possibilities. Plant Methods 17:83.

10. Klement Z. 1963. Rapid Detection of the Pathogenicity of Phytopathogenic Pseudomonads. 4890. Nature 199:299–300.

**Supplemental figure captions:**

Fig S1. Disease severity on ‘California wonder’ pepper seedlings after 14-days post-inoculation (dpi) with 16 *Pseudomonas syringae* pv. *syringae* (*Pss*) strains. Disease severity is represented by the number of lesions on two lower leaves of infected seedlings at 14dpi. The experiment was conducted twice with a minimum of four replicates for each strain at different time points and data from the two experiments were combined. The black bars represent the median no. of lesions or log (CFU/g) and the letters represent strains that are significantly connected (P<0.05).

Fig S2. Disease incidence on ‘California Wonder’ pepper seedlings at 3-, 7- and 14-days post-inoculation (dpi) with 16 *Pseudomonas syringae* pv. *syringae* (*Pss*) strains. Disease incidence was recorded as the percentage of seedlings showing any *Pss* symptoms at each timepoint. The experiment was conducted twice with a minimum of four replicates for each strain at different time points and data from the two experiments were combined. The letters represent strains that are significantly connected (P<0.05). Colored bars represent disease incidence at 3 dpi (blue), 7 dpi (red), and 14 dpi (green).

Fig. S3. Antimicrobial resistance genes of *Pseudomonas syringae*pv*. syringae* (*Pss*) strains that are part of the core and variable genome. A total of 27 antibiotic resistance genes were identified with only 6 variable antimicrobial resistance genes between the *Pss* strains. The light grey boxes indicate absence of the gene and dark grey boxes indicate the presence of genes with darker grey/ black boxes indicating more than one copy of the gene.

Fig. S4. Motility genes of *Pseudomonas syringae*pv*. syringae* (*Pss*) strains (n=16) that are part of the core and variable genome. A total of 87 motility genes were identified with only 2 variable motility genes between the *Pss* strains. The light grey boxes indicate absence of the gene and dark grey boxes indicate the presence of genes with darker grey/ black boxes indicating more than one copy of the gene.

Fig. S5. Biofilm genes of *Pseudomonas syringae*pv*. syringae* (*Pss*) strains (n=16) that are part of the core and variable genome. A total of 30 biofilm genes were identified with only 2 variable biofilm genes between the *Pss* strains. The light grey boxes indicate absence of the gene and dark grey boxes indicate the presence of genes with darker grey/ black boxes indicating more than one copy of the gene.

Fig. S6. Secretion system genes of *Pseudomonas syringae*pv*. syringae* (*Pss*) strains (n=16) that are part of the core and variable genome. (A) A total of 94 secretion system genes were identified. (B) A total of 29 secretion system genes were variable between the *Pss* strains. The light grey boxes indicate absence of the gene and dark grey boxes indicate the presence of genes with darker grey/ black boxes indicating more than one copy of the gene.

Fig. S7. Variable genes correlated with disease severity at 3- and 7-days post infection among *Pss* strains. The red cells in the heatmap indicate gene absence and blue cells indicate gene presence. The yellow to purple colors in the boxes at the top represents high to low disease severity, and strains are ordered by disease severity.

Fig. S8. Significant SNPs in virulence genes (biofilm, motility, and secretion system) correlated with disease severity at 3- and 7-days post infection (dpi; r^2^ of 0.498 to 0.779; P<0.05) between the *Pseudomonas syringae*pv*. syringae* (*Pss*) strains. The red boxes indicate absence of the gene and blue boxes indicate the presence of genes. “Chrom” and “pos” indicate the chromosome location and position of the SNPs, respectively.

**Supplemental table captions:**

Table S1. Year and location of isolation from peppers, LOPAT and PCR results for *Pseudomonas syringae* pv*. syringae (Pss*) strains used in the study. All strains were grown in M9 minimal broth or NBY agar plates at 28˚C for 24 h. The LOPAT tests placed all isolates in the *P. syringae* group. The PCR tests with two pathovar *syringae*-specific primers (*syrB* and *hrpZ*) identified all 16 strains as *Pss*. All strains were sourced from the Miller lab collection. (+) indicates positive result and (-) indicates negative result for each experiment.

Table S2. Correlated genes detected by the multivariate correlation analysis and Scoary between the variable genome and in vitro characteristic (biofilm, motility, and growth). A Benjamini-Hochberg correction was used to determine significantly associated genes (P<0.05 and odds ratio>1). Naïve_p indicated the significant p-value prior to the Benjamini-Hochberg correction. Benjamini-Hochberg correction with P > 0.05 indicates insignificantly correlated genes.

Table S3. *Pseudomonas syringae* strains (n=18) both within and outside the P. syringae pv. syringae phylogenetic group, obtained from NCBI for the phylogenetic analysis. The host of isolation, phylogroup and reference for each strain is indicated in the columns.

Table S4. Variable genes (n=812) within the 16 *Pseudomonas syringae* pv*. syringae (Pss*) strains. The presence of a gene is indicated by “1” whereas the absence of a gene is indicated by “0” for each strain.

Table S5. Multiple testing correction for all the variable genes (n=142 and 151) that were positively or negatively correlated (r2 of ± 0.5 to 0.675) with disease severity (3- and 7-days post infection (dpi)) of *Pseudomonas syringae* pv*. syringae* (*Pss*) strains. None of the listed genes were significantly correlated with disease severity (P >0.05) after Benjamini-Hochberg correction. Benjamini-Hochberg correction with P > 0.05 indicates insignificantly correlated genes.

Table S6. Pseudogenes (n=184) present in the variable genome of the 16 *Pseudomonas syringae* pv*. syringae* (*Pss*) strains. The numbers (0 to 9) indicate the number of pseudogenes present in each gene carried by the different strains.

Table S7. Multiple testing correction for SNPs within virulence genes (biofilm, motility, and secretion system) correlated with disease severity (3- and 7-days post infection (dpi)) of *Pseudomonas syringae* pv*. syringae* (*Pss*) strains. None of the listed genes were significantly correlated with disease severity (P >0.05) after Benjamini-Hochberg correction. Benjamini-Hochberg correction with P > 0.05 indicates insignificantly correlated genes.

Table S8. Pathovar-specific primers used in PCR assays to identify *Pseudomonas syringae* pv. *syringae* (*Pss*) strains from peppers. Two sets of primers (*hrpZ* and *syrB*) were used at appropriate annealing temperatures.
