# Supplementary material for: In vitro, in planta, and comparative genomic analyses of Pseudomonas syringae pv. syringae strains of pepper (Capsicum annuum var. annuum)
Source: Microbiol Spectr. 2024 May 7;12(6):e00064-24. doi: 10.1128/spectrum.00064-24 (PMC11237606; doi:10.1128/spectrum.00064-24)
Supplement: Table S1 — Year and location of isolation from peppers, LOPAT and PCR results. [file spectrum.00064-24-s0003.docx]

| *Pss* strains | | | LOPAT test for *P.syringae* identification | | | | | PCR for *Pss* identification | |
| --- | --- | --- | --- | --- | --- | --- | --- | --- | --- |
| *Pss* ID | County | Year | Levan production | Oxidase activity | Pectolytic activity | Arginine dihydrolase | Tobacco HR | syrB | hrpZ |
| SM914-13 | Sandusky | 2013 | + | - | - | - | + | + | + |
| SM1038-14 | Wayne | 2014 | + | - | - | - | + | + | + |
| SM109-18 | Wayne | 2018 | + | - | - | - | + | + | + |
| SM155-18 | Wayne | 2018 | + | - | - | - | + | + | + |
| SM156-18 | Sandusky | 2018 | + | - | - | - | + | + | + |
| SM51-19 | Wayne | 2019 | + | - | - | - | + | + | + |
| SM1030-14 | Wayne | 2014 | + | - | - | - | + | + | + |
| SM1042-14R | Wayne | 2014 | + | - | - | - | + | + | + |
| SM04-2018-04 | Wayne | 2018 | + | - | - | - | + | + | + |
| SM1031-14 | Wayne | 2014 | + | - | - | - | + | + | + |
| SM191-1 | Seneca | 2021 | + | - | - | - | + | + | + |
| SM205-2 | Seneca | 2021 | + | - | - | - | + | + | + |
| SM181-4 | Seneca | 2021 | + | - | - | - | + | + | + |
| SM190-8 | Seneca | 2021 | + | - | - | - | + | + | + |
| SM207-3 | Sandusky | 2021 | + | - | - | - | + | + | + |
| SM226-1 | Wayne | 2021 | + | - | - | - | + | + | + |

Table S1. Year and location of isolation from peppers, LOPAT and PCR results for *Pseudomonas syringae* pv. *syringae* (*Pss*) strains used in the study. All strains were grown in M9 minimal broth or NBY agar plates at 28˚C for 24 h. The LOPAT tests placed all isolates in the *P. syringae* group. The PCR tests with two pathovar syringae-specific primers (syrB and hrpZ) identified all 16 strains as Pss. All strains were sourced from the Miller lab collection. (+) indicates positive result and (-) indicates negative result for each experiment.
