# Supplementary material for: In vitro, in planta, and comparative genomic analyses of Pseudomonas syringae pv. syringae strains of pepper (Capsicum annuum var. annuum)
Source: Microbiol Spectr. 2024 May 7;12(6):e00064-24. doi: 10.1128/spectrum.00064-24 (PMC11237606; doi:10.1128/spectrum.00064-24)
Supplement: Table S8 — Pathovar-specific primers used in PCR assays to identify Pseudomonas syringae pv. syringae (Pss) strains from peppers. [file spectrum.00064-24-s0010.docx]

| Primer name | Sequence | GC | Annealing Tm | Expected band size |
| --- | --- | --- | --- | --- |
| PsshrpZ2f | CGCTTCAGGAAGTTGTCGTG | 55% | 56.3˚C | 330bp |
| PsshrpZ2r | GCATCGGCATATCGTCTTCG | 55% | 55.8˚C |  |
| syrB1 | CTTTCCGTGGTCTTGATGAGG | 52.4% | 55.5˚C | 752 bp |
| syrB2 | TCGATTTTGCCGTGATGAGTC | 47.6% | 55.5˚C |  |

Table S8. Pathovar-specific primers used in PCR assays to identify Pseudomonas syringae pv. syringae (Pss) strains from peppers. Two sets of primers (hrpZ and syrB) were used at appropriate annealing temperatures.
